# Long non-coding RNA HUMT hypomethylation promotes lymphangiogenesis and metastasis via activating FOXK1 transcription in triple-negative breast cancer

**DOI:** 10.1186/s13045-020-00852-y

**Published:** 2020-03-05

**Authors:** Shaoquan Zheng, Lu Yang, Yutian Zou, Jie-ying Liang, Peng Liu, Guanfeng Gao, Anli Yang, Hailin Tang, Xiaoming Xie

**Affiliations:** 1 Department of Breast Oncology, Sun Yat-sen University Cancer Center, 651 East Dongfeng Road, Guangzhou, 510060 China; 2 Department of Medical Oncology, Sun Yat-sen University Cancer Center, 651 East Dongfeng Road, Guangzhou, 510060 China; 3 State Key Laboratory of Oncology in South China, Collaborative Innovation Center for Cancer Medicine, Sun Yat-sen University Cancer Center, 651 East Dongfeng Road, Guangzhou, 510060 Guangdong China

**Keywords:** Triple-negative breast cancer, Lymph node metastasis, Long non-coding RNA, Y-box binding protein 1, Forkhead box k1

## Abstract

**Background:**

Triple-negative breast cancer (TNBC) is the most malignant subtype of breast cancer with highly invasive ability and metastatic nature to the lymph nodes. Long non-coding RNAs (lncRNAs) have been widely explored in cancer tumorigenesis and progression. However, their roles in TNBC lymph node metastasis remains rarely studied.

**Methods:**

The expression of lncRNA highly upregulated in metastatic TNBC (HUMT) in cell lines and tissues was detected by quantitative real-time PCR (qRT-PCR) and in situ hybridization (ISH). RNA immunoprecipitation (RIP) and RNA pulldown were used to verify the interaction between lncRNA and protein. Chromatin immunoprecipitation (CHIP) and dCas9-gRNA-guided chromatin immunoprecipitation (dCas9-CHIP) were conducted to identify the specific binding site of HUMT-YBX1 complex. Western blot was used to detect the downstream of HUMT.

**Results:**

HUMT was significantly upregulated in lymph node invasive cells and predicted poorer clinical prognosis. Functional study indicated that HUMT promoted lymphangiogenesis and lymph node metastasis. Bioinformatic analysis and qRT-PCR showed that the high expression of HUMT was correlated with the hypomethylation status of its promoter region. Further, HUMT recruited Y-box binding protein 1 (YBX1) to form a novel transcription complex and activated the expression of forkhead box k1 (FOXK1), thus enhancing the expression of vascular endothelial growth factor C (VEGFC). The therapeutic value was further validated in patient-derived xenograft (PDX) models, and a combined marker panel exhibited a better prognostic value for TNBC in receiver operating characteristic (ROC) analysis.

**Conclusions:**

Our study identified a novel TNBC lymph node metastasis-associated lncRNA, which promoted TNBC progression and indicated a novel biomarker and potential therapeutic target for TNBC lymph node metastasis.

## Introduction

Breast cancer is the most common cancer among women worldwide with increasing incidence [1, 2]. Although triple-negative breast cancer (TNBC) is associated with poorer clinical outcomes and fewer treatments than any other molecular subtype, surgery and chemotherapy still remain as the only first-line regimens [3]. Neither endocrine therapy nor routine targeted therapy was effective on TNBC, such as tamoxifen and trastuzumab. Although representing only about 10–20% of breast cancers, TNBC is more invasive and malignant with a higher rate of lymph node metastasis and exhibited shorter median survival [4, 5]. Hence, it is urgent to investigate novel biomarkers and molecular mechanisms of lymph node metastasis, which was generally considered as the first step of cancer cells.

In recent years, epigenetic events are considered as crucial factors in tumorigenesis and progression. The development and progression of breast cancer consist of various genetic and epigenetic changes [4, 6]. Dynamic and reversible DNA methylation status provides potential promising therapeutic targets for cancers [7, 8].

Long non-coding RNAs (lncRNAs) are a special class of transcripts with a length of more than 200 bases and well known for their limited protein-coding potential [9, 10]. Previous studies have identified a series of lncRNAs involved in multilevel regulation of gene expression, including transcription regulation by affecting DNA methylation or transcription factor activity. Besides, lncRNAs also function in post-transcription regulation, such as endogenous competing RNA and protein stabilizer [11–18]. Although many lncRNAs have been reported to be aberrantly expressed in breast cancer [19] and involved in hallmarks of cancer, including apoptosis [20], proliferation [21], metabolism [22], and metastasis [23], the functions and mechanisms of most lncRNAs in triple-negative breast cancer lymph node metastasis and immune response still remain unknown [24, 25].

In this study, we investigated the epigenetic alteration and biological effect of a novel triple-negative breast cancer lymph node-associated lncRNA HUMT (lncRNA highly upregulated in metastatic TNBC-lymph node, LINC00857), on cancer cell proliferation and metastasis. Microarray analysis indicated that HUMT was significantly upregulated in highly lymph node invasive cells. Further mechanistic study revealed that HUMT expression was regulated in an epigenetic way, and it recruited the YBX1 protein to form the transcription complex on the FOXK1 promoter region and enhanced its transcription, resulting in breast cancer proliferation and lymph node metastasis. Interestingly, our results showed that HUMT might also inhibit the recruitment and activation of NK cells in the microenvironment. Our data revealed a novel mechanism for lymph node metastasis in TNBC, suggesting that HUMT could be a potential therapeutic target.

## Material and methods

### Study subjects

Clinical samples were collected from patients who underwent breast cancer resection in Sun Yat-sen University Cancer Center (SYSUCC). Detailed information for the sample collection was provided in Supplementary Material and Methods (Additional file 10).

### Plasmid constructions and transfections

Vectors for overexpressing, dCas9-CHIP, CRISPR were constructed, and details were provided in Supplementary Material and Methods. Primers, siRNAs, and sgRNAs were listed (Additional files 13 and 14).

### In vitro experiments

Full detailed procedures of cell cultures, cell viability, migration and invasion assay, endothelial cell tube formation assay, western blot, quantitative real-time PCR (qRT-PCR), RNA immunoprecipitation (RIP), RNA pulldown, and chromatin immunoprecipitation (CHIP) are provided in Supplementary Material and Methods.

### In situ hybridization, fluorescence in situ hybridization, and immunohistochemistry

In situ hybridization (ISH), fluorescence in situ hybridization (FISH), and immunohistochemistry (IHC) were performed as previously described, and details are provided in Supplementary Material and Methods [14, 26].

### Animal experiments

All the in vivo experiments were performed following institutional and international guidelines and regulations. Detailed procedures were provided in Supplementary Material and Methods.

### Bioinformatic and statistical analysis

Corresponding methods and tools for the analysis of PAR-CLIP, CHIP-seq, and CIBERSORT are provided in supplementary tables (Additional files 15 and 16). Student’s *t* test and *χ*^2^ test were used for comparison of the results between two groups, and the non-parametric test was adopted for data in abnormal distribution. One-way ANOVA was used for comparison in more than two groups. The Spearman test was adopted for analyzing the correlation of HUMT/YBX1/FOXK1. The Kaplan-Meier method with a log-rank test was used to compare the overall survival (OS) and disease-free survival (DFS) between the groups. The receiver operating characteristic (ROC) curve was adopted for analyzing the diagnostic value of HUMT in patients with TNBC. Statistical analysis was performed on GraphPad Prism 7 (GraphPad), MedCalc 18.2.1 software (MedCalc), and R 3.5.3 software (https://www.r-project.org). The results were presented as mean ± SEM. A *P* value of < 0.05 was determined as statistically significant, and all *P* values were two-tailed.

## Results

### HUMT expression is significantly upregulated in cancer cells with lymph node-invasive nature and predicted poor prognosis

**Fig. 1:**
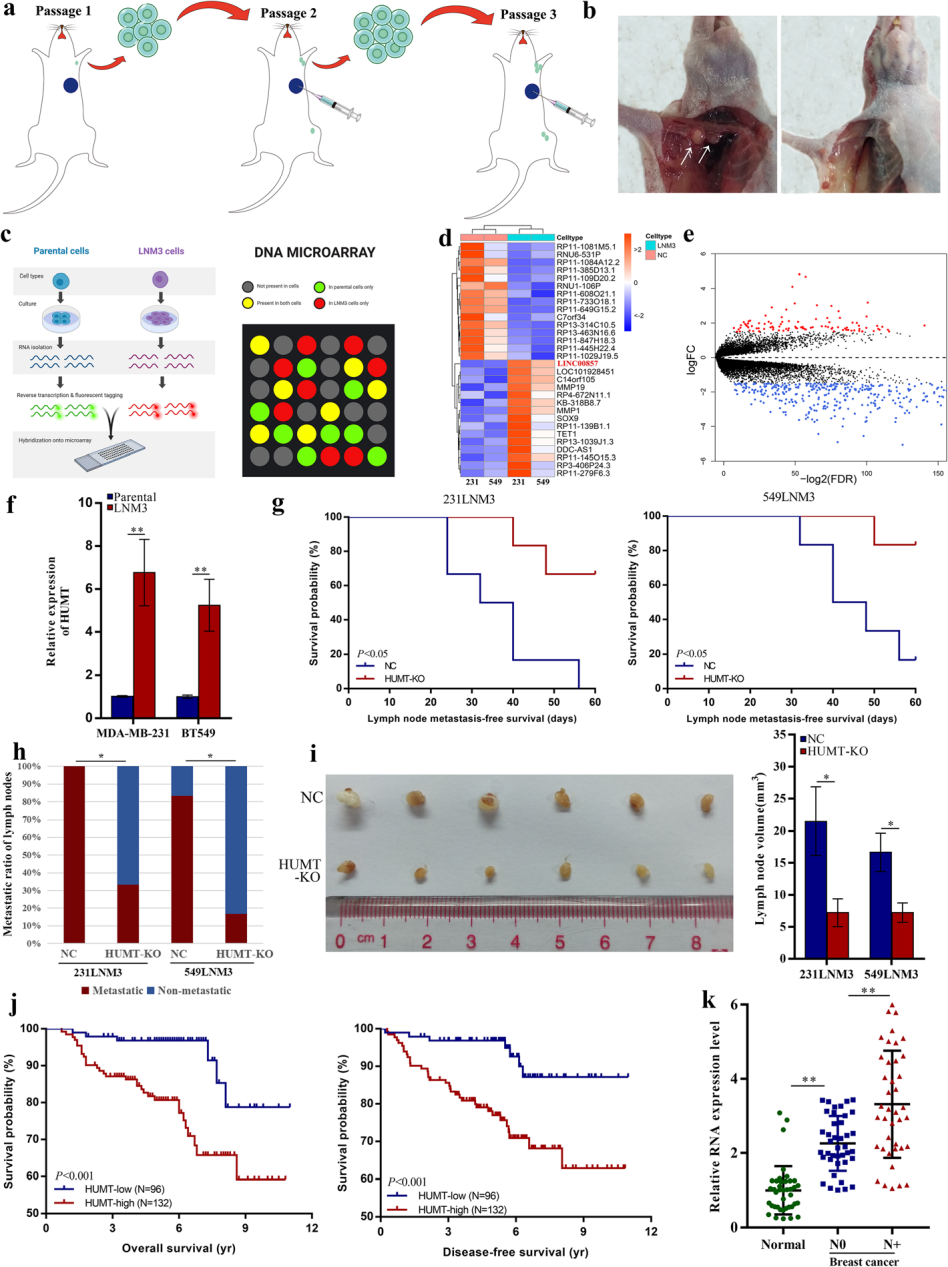
HUMT is responsible for triple-negative breast cancer lymph node metastasis. **a** The establishment of triple-negative cell lines with highly lymph node-invasive ability in mice. **b** Representative picture for LNM3 (left) and parental (right) cells with different lymph node-invasive ability in mice. **c** Schematic procedure of microarray detection of LNM3 and parental cells. **d** Heatmap and **e** volcano plot profiling identified the top significant RNA alteration. **f** The upregulation of HUMT in LNM3 cells was further validated by qRT-PCR. **g** Lymph node metastasis-free survival analysis and **h** metastatic lymph node ratio of mice injected with modulated 231LNM3 and 549LNM3 cells. **i** The representative picture and the corresponding volume of lymph nodes in different groups (231LNM3). **j** Kaplan-Meier analysis indicated a better OS and DFS in patients with low HUMT expression. **k** qRT-PCR identified a significantly higher level of HUMT in primary TNBC with lymph node metastasis compared with paracancerous tissue and primary TNBC without lymph node metastasis. Data were shown as mean ± SD; *P < 0.05; **P < 0.01

To investigate the biological mechanism of TNBC lymph node metastasis, we grafted MDA-MB-231 and BT549 cells into the fat pads of nude mice and performed passages to screen highly lymph node-invasive cells in vivo (Fig. 1a, b). Cancer cells in metastatic lymph node from the third passage displayed a highly invasive nature to lymph nodes. Highly invasive cells in metastatic lymph nodes were isolated and termed 231LNM3 and 549LNM3.

To identify which biological change exerted an impact on triple-negative breast cancer lymph node metastasis, highly lymph node-invasive cells and parental cells were applied to genome-wide microarray in replicates (Fig. 1c). Representative upregulated and downregulated transcripts were shown in the heatmap (Fig. 1d) and volcano plot (Fig. 1e). HUMT was significantly upregulated in LNM3 cells compared with parentals and further validated by qRT-PCR (Fig. 1f).

We firstly evaluated the coding potential of HUMT using NCBI ORFfinder. No typical protein-coding open reading frame (ORF) (> 300 nt) was found on the sequence of HUMT, and there is a very low probability for the coding potential of HUMT (Additional file 1: Fig. S1a, b). The result was further validated by a PhyloCSF model and coding potential assessment tools (CPAT) (Additional file 1: Fig. S1c). The PhyloCSF value along the entire sequence of HUMT was calculated, and the negative value suggested a low coding potential. We further used the CPAT to confirm this result (Additional file 1: Fig. S1d). In summary, these observations confirmed that HUMT did not have a coding capacity.

The bioinformatic analysis also showed that HUMT was significantly upregulated in tumors compared with paired normal tissues of TNBC in the TCGA cohort. A pan-cancer analysis confirmed a higher level of HUMT in tumor tissue of various cancers (Additional file 2: Fig. S2).

Then, we conducted in vivo experiments on nude mice to validate the function of HUMT. The lymph node metastasis model using 231LNM3 and 549LNM3 with normal control (NC) or HUMT-KO (knock-out) was constructed. The HUMT-KO group exhibited improved lymph node metastasis-free survival (LNMFS) compared with the controls (Fig. 1g), a lower rate of lymph node metastasis, and smaller metastatic lymph node volume (Fig. 1h, i), indicating that HUMT promoted lymph node metastasis in TNBC.

Furthermore, the expression level of HUMT was detected in 228 cases of TNBC from SYSUCC. The patients were divided into two groups: HUMT-low and HUMT-high. The association between HUMT expression and clinicopathological characteristics was analyzed. A crosstabs analysis showed that HUMT expression was significantly correlated with the AJCC T stage and N stage (Additional file 11: Table S1).

The Kaplan-Meier method with a log-rank test showed a significantly poorer overall survival (OS) and disease-free survival (DFS) in the HUMT-high group (Fig. 1j). Moreover, the qRT-PCR analysis showed that a significantly higher level of HUMT in primary TNBC with lymph node metastasis compared with paracancerous tissues and those without lymph node metastasis, suggesting that HUMT might serve as a predictor for lymph node metastasis in TNBC (Fig. 1k).

Taken together, HUMT was correlated with lymph node metastasis and predicted tumor progression in TNBC.

### HUMT promotes triple-negative breast cancer proliferation, metastasis, and lymphangiogenesis in vitro

**Fig. 2:**
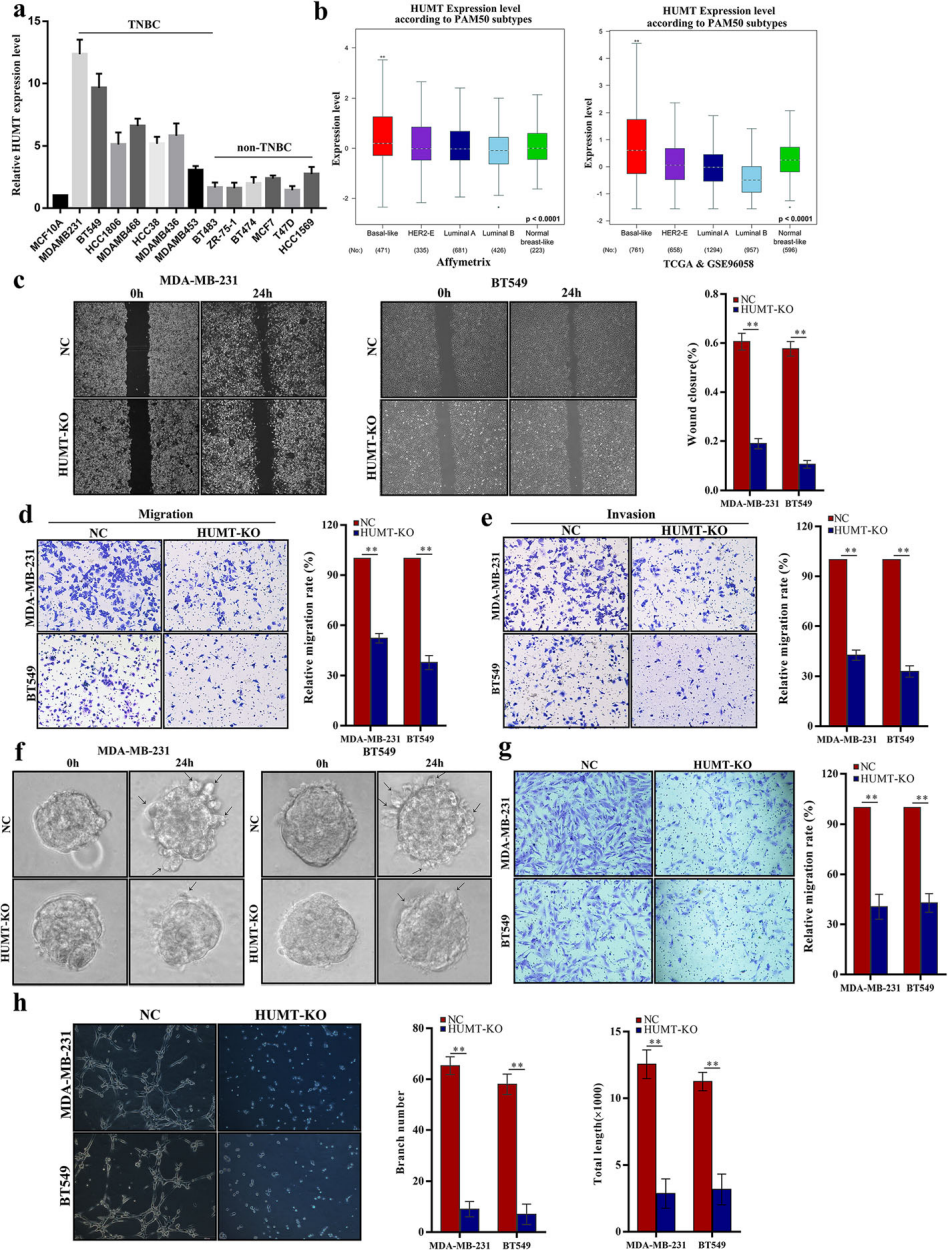
HUMT regulates lymph node metastasis of triple-negative breast cancer cells in vitro. **a** HUMT expression in breast cancer cell lines. MCF10A represents normal breast epithelial cells. **b** HUMT was more highly expressed in the basal-like subtype in a comprehensive analysis of the GEO datasets and TCGA database. **c** Representative graphs (left) and quantification (right) of wound healing assay. Representative graphs (left) and quantification (right) of Transwell migration (**d**) and invasion **e** assay in modulated MDA-MB-231 and BT549 cells. **f** Representative graphs of 3D invasion assay in modulated MDA-MB-231 and BT549 cells. **g** Representative picture (left) and quantification (right) of HLEC migration assay. **h** Representative graphs (left) and branch number and total tube length quantification (right) of HLEC tube formation assay cultured with medium supernatant from NC or HUMT-KO-modulated MDA-MB-231 and BT549 cells. Data were shown as mean ± SD; **P < 0.01

We further investigated the RNA levels of HUMT in breast cancer cell lines and normal mammary epithelial cells using qRT-PCR. Interestingly, the expression level of HUMT was significantly higher in triple-negative breast cancer cell lines, especially MDA-MB-231 and BT549, than non-TNBC cell lines and normal epithelial cells (Fig. 2a). Further bioinformatic analysis confirmed a higher expression level of HUMT in basal-like patients than non-basal like ones, indicating HUMT might be responsible for the malignant characteristics of TNBC (Fig. 2b).

As the lymph node metastasis is always positively correlated with tumor cell proliferation and metastatic nature, we constructed the HUMT-KO MDA-MB-231 and BT549 cell lines using CRISPR methods to further investigate whether HUMT was involved in cell proliferation, metastasis, and lymphangiogenesis. CCK8 assay showed that cell proliferation was significantly suppressed in HUMT-KO cells than controls (Additional file 3: Fig. S3a). This was further validated in colony formation assay as the colony-forming capacity was significantly inhibited in the HUMT-KO group (Additional file 3: Fig. S3b). Wound healing assay, Transwell migration, and invasion assay indicated that HUMT silencing significantly suppressed the migration ability of cancer cells (Fig. 2c–e). Furthermore, a 3D invasion assay was conducted and showed a consistent result for invasion ability (Fig. 2f).

The migration activity of HLECs could be significantly impacted in the culture medium supernatant of HUMT-KO cells compared with controls, indicating a crucial role of HUMT in triple-negative breast cancer lymph node metastasis (Fig. 2g). Moreover, we examined whether HUMT promoted lymphangiogenesis by studying its effect on the tube formation ability of HLECs, which is a critical process during breast cancer lymph node metastasis. The culture medium supernatant of HUMT-KO cells significantly abolished the tube formation capacity of HLECs as the branch number and total length of tubes were decreased compared with the control groups (Fig. 2h).

In summary, these results indicated that HUMT promotes proliferation, lymphangiogenesis, and lymph node metastasis in TNBC in vitro.

### HUMT upregulation is due to promoter hypomethylation

**Fig. 3:**
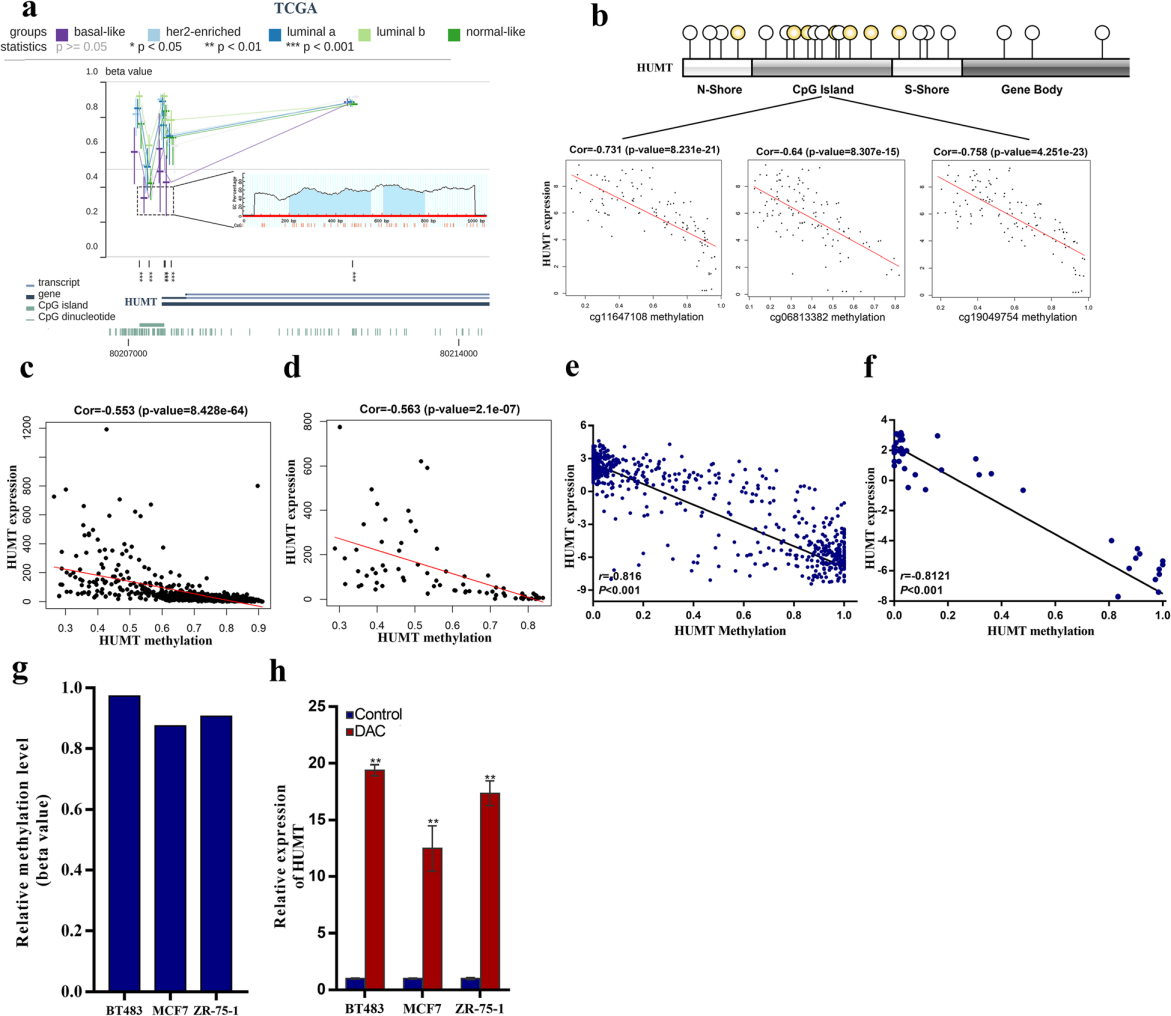
HUMT expression was regulated in an epigenetic way. **a** Bioinformatic analysis indicated CpG enrichment in the promoter region, and patients with basal-like subtype exhibited a lower HUMT methylation level. **b** HUMT expression was significantly correlated with a methylation probe signal located in the CpG island of the promoter region. **c**, **d** Overall DNA methylation level of HUMT was significantly correlated with RNA expression in patients with breast cancer and TNBC of the TCGA database. **e**, **f** Overall DNA methylation level of HUMT was significantly correlated with RNA expression in all cell lines and breast cancer cell lines of the CCLE database. **g** Representative hypermethylation status in BT483, MCF-7, and ZR-75-1 cells (CCLE). **h** A methyltransferase inhibitor, DAC, could significantly upregulate the expression level of HUMT in BT483, ZR-75-1, and MCF-7 cells. Data were shown as mean ± SD; *P < 0.05; **P < 0.01; **P < 0.001

To further elucidate the mechanism of HUMT upregulation, we analyzed the correlation between DNA methylation and the expression level of HUMT as DNA methylation was one of the most common ways of epigenetic modulation for gene expression [19, 27, 28]. Bioinformatic analysis indicated that basal-like breast cancer patients harbored the lowest level of DNA methylation at the promoter region in breast cancer subtypes, which was consistent with the highest RNA expression level mentioned above. Besides, two GC-enriched regions were also predicted and indicated CpG islands in the promoter region of HUMT (Fig. 3a). The analysis of CpG island-specific methylation revealed a significant negative correlation between methylation and expression levels in The Cancer Genome Atlas (TCGA) Program database (Fig. 3b). We further analyzed the whole-gene DNA methylation level of breast cancer patients in TCGA. The results showed a significant negative correlation between DNA methylation level and expression level of HUMT in all breast cancer (*r* = − 0.553, *P* < 0.001) and TNBC (*r* = − 0.563, *P* < 0.001) (Fig. 3c, d). Moreover, the methylation status of HUMT in Cancer Cell Line Encyclopedia (CCLE) was analyzed and revealed a significantly negative correlation in all cancer cell lines (*r* = − 0.816, *P* < 0.001) and breast cancer cell lines (*r* = − 0.812, *P* < 0.001) (Fig. 3e, f).

BT483, MCF7, and ZR-75-1 cells were identified as the cell lines with hypermethylation status at the promoter region of HUMT (Fig. 3g). The qRT-PCR analysis was conducted after decitabine (DAC) treatment. In vitro experiments showed that decitabine, a methyltransferase inhibitor, could significantly upregulate the expression of HUMT in MCF7, ZR-75-1, and BT483 cells, indicating that HUMT was regulated in an epigenetic way (Fig. 3h). Pan-cancer analysis of TCGA datasets also revealed a significant correlation between HUMT expression and methylation level of CpG island in the promoter region (Additional file 4: Fig S4).

Taken together, HUMT was upregulated in highly invasive cells due to the hypomethylation at the promoter region.

### HUMT regulates FOXK1 expression by recruiting YBX1 to form a transcription complex

We next explored the mechanism by which HUMT regulates cancer progression in TNBC. As the functions of lncRNAs were tightly associated with subcellular distribution, bioinformatic analysis by lncATLAS was used to predict the location. Nearly half of HUMT was predicted to be located in the nuclei in cell lines (Additional file 5: Fig. S5a). We further extracted the RNA in the cytoplasm and nuclei. qRT-PCR showed that about 40% of HUMT was distributed in the nuclei, which was further confirmed by FISH (Additional file 5: Fig. S5b-c).

**Fig. 4:**
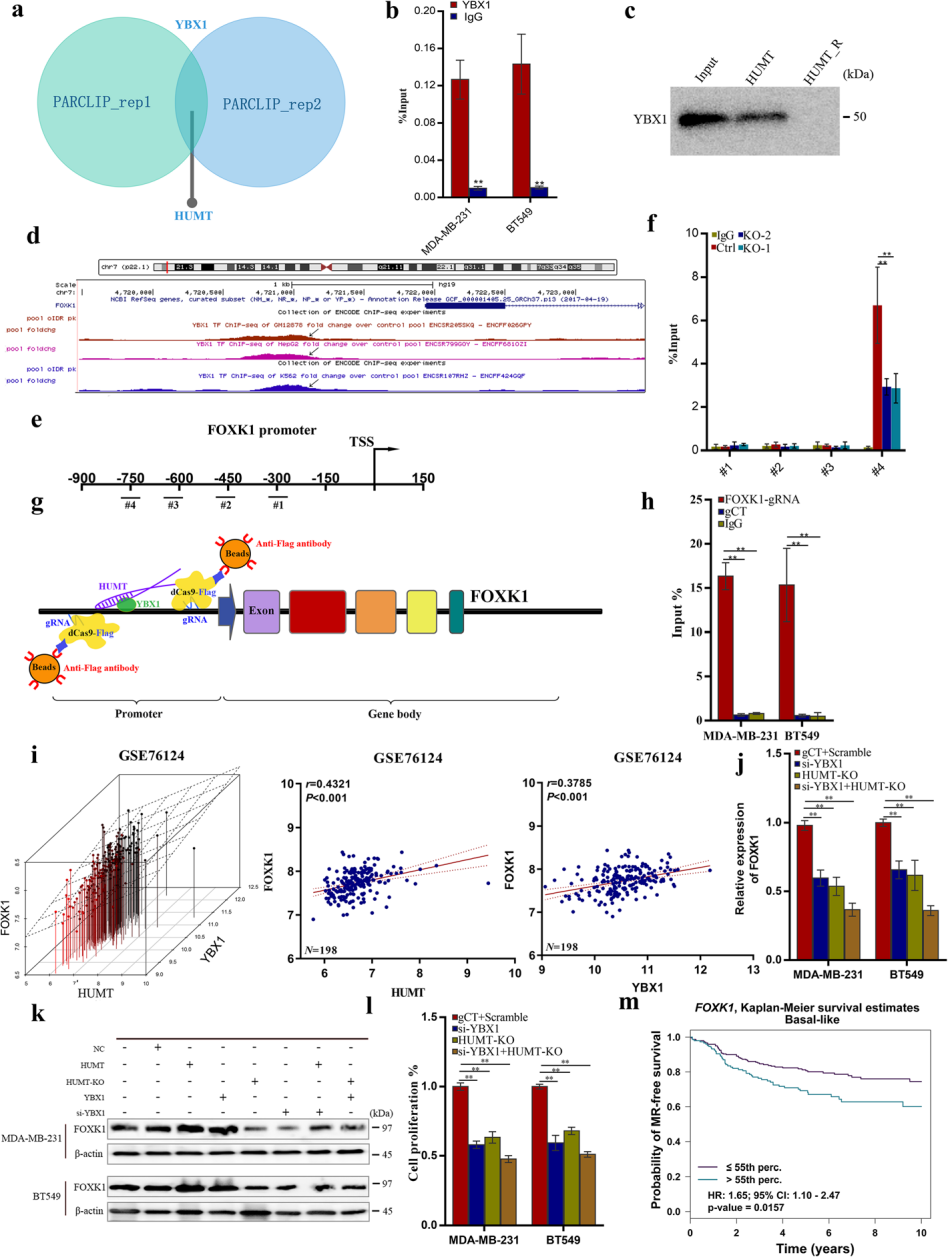
HUMT interacted with YBX1 to regulate FOXK1 expression and cell proliferation. **a** Venn diagram showed that HUMT interacted with YBX1 using PAR-CLIP in two independent biological replicates (GSE133620). **b** RNA immunoprecipitation (RIP) using the YBX1 antibody followed by qRT-PCR for HUMT. **c** Validation of the interaction between HUMT and YBX1 by RNA pulldown assay. HUMT antisense biotinylated probes were used as the negative control. **d** CHIP-seq identified YBX1 binding peaks on the promoter region of FOXK1 in three independent cells (ENCODE). **e** Schematic illustration of the PCR-amplified region of FOXK1 promoter. **f** Chromatin immunoprecipitation (CHIP) was performed using the YBX1 antibody to identify the YBX1-binding regions on the FOXK1 promoter. IgG was used as a negative control. **g** Schematic summary of the dCas9-CHIP. A 3xFLAG-dCas9-HA-2xNLS fusion protein (FLAG-dCas9) consisting of an N-terminal triple FLAG (3xFLAG) epitope tag and catalytically inactive Cas9 endonuclease (dCas9) was expressed with guide RNAs (gRNAs) in appropriate cell context to validate HUMT interaction with FOXK1 promoter. **h** Enrichment of HUMT on the FOXK1 promoter region. IgG and scrambled gRNA were used as negative controls. **i** Correlation between YBX1, HUMT, and FOXK1 RNA expression in GSE76124. **j** FOXK1 expression in YBX1-KD and HUMT-KO cells was detected by qRT-PCR. **k** FOXK1 expression in YBX1 and HUMT modulated cells by western blot. **l** Cell proliferation in YBX1-KD and HUMT-KO cells. **m** Comprehensive analysis of basal-like breast cancer in GEO datasets using bc-GenExMiner-identified FOXK1 to be a prognostic factor for metastatic relapse-free survival. Data were shown as mean ± SD; *P < 0.05; **P < 0.01

Previous studies have reported the important role of lncRNAs in transcription by recruiting corresponding proteins [29, 30]. A considerable part of HUMT was located in the nucleus of the cancer cells, indicating that HUMT might exert its function by recruiting transcription complexes and further enhance or inhibit gene transcription as previously reported. To verify this hypothesis and identify HUMT-interacting proteins, we utilized a GEO dataset and forecasted that a DNA binding protein YBX1 might bind to HUMT (Fig. 4a). The interaction of YBX1 protein with HUMT was validated using RNA immunoprecipitation (RIP) assay (Fig. 4b). RNA pulldown using a biotinylated RNA probe of HUMT was used as a reverse proof (Fig. 4c).

Previous studies reported that YBX1 played a crucial role in transcription [31, 32]. To further investigate whether the HUMT-YBX1 complex exerted its function by transcription regulation, we analyzed the CHIP-seq result of YBX1 protein in three independent cell lines from the ENCODE database to identify candidate DNA with which YBX1 might interact (Additional file 6: Fig. S6a-c). FOXK1, a transcript well known for functions related to extensive cancer progression activity, drew our attention. The binding motif of YBX1 on the FOXK1 promoter region could be predicted (Additional file 6: Fig. S6d). Further analysis revealed the YBX1-binding peaks about 800 bp upstream TSS, and specific primers for different promoter segments of FOXK1 were designed (Fig. 4d, e). To determine which site(s) YBX1 bound within the FOXK1 promoter, ChIP was performed. Our results revealed that YBX1 protein bound to FOXK1 at the − 700 to approximately − 850-bp promoter region of FOXK1, and the combination was significantly downregulated when HUMT was knocked out (Fig. 4f). Next, we conducted a dCas9-CHIP assay to further validate the occupancy of HUMT at FOXK1 promoter region (Fig. 4g). HUMT was significantly enriched in the group using FOXK1 promoter-targeted gRNAs than IgG or control gRNA group (Fig. 4h).

Next, we investigated the effect of the HUMT-YBX1 complex on the FOXK1 expression and cell proliferation. Bioinformatic analysis showed that the expression of HUMT and YBX1 positively correlated with FOXK1 (Fig. 4i). HUMT-KO or YBX1-KD could significantly decrease the FOXK1 expression level detected by qRT-PCR (Fig. 4j). Moreover, western blot confirmed that FOXK1 was upregulated following YBX1 or HUMT overexpression, and these effects could be partly reversed by HUMT or YBX1 silencing (Fig. 4k). In addition, we found that HUMT-KO and YBX1-KD significantly suppressed cell proliferation of MDA-MB-231 and BT549 (Fig. 4l). Further bioinformatic analysis from the Breast Cancer Gene-Expression Miner (bc-GenExMiner v4.3) showed that FOXK1 predicted a poorer metastasis-free survival in patients with basal-like breast cancer, which was consistent with our results (Fig. 4m). Together, these data demonstrated that HUMT, YBX1, and FOXK1 could form a transcription complex, and HUMT could regulate the FOXK1 expression by recruiting YBX1.

### FOXK1 is crucial to HUMT-mediated cell proliferation, metastasis, and lymphangiogenesis

**Fig. 5:**
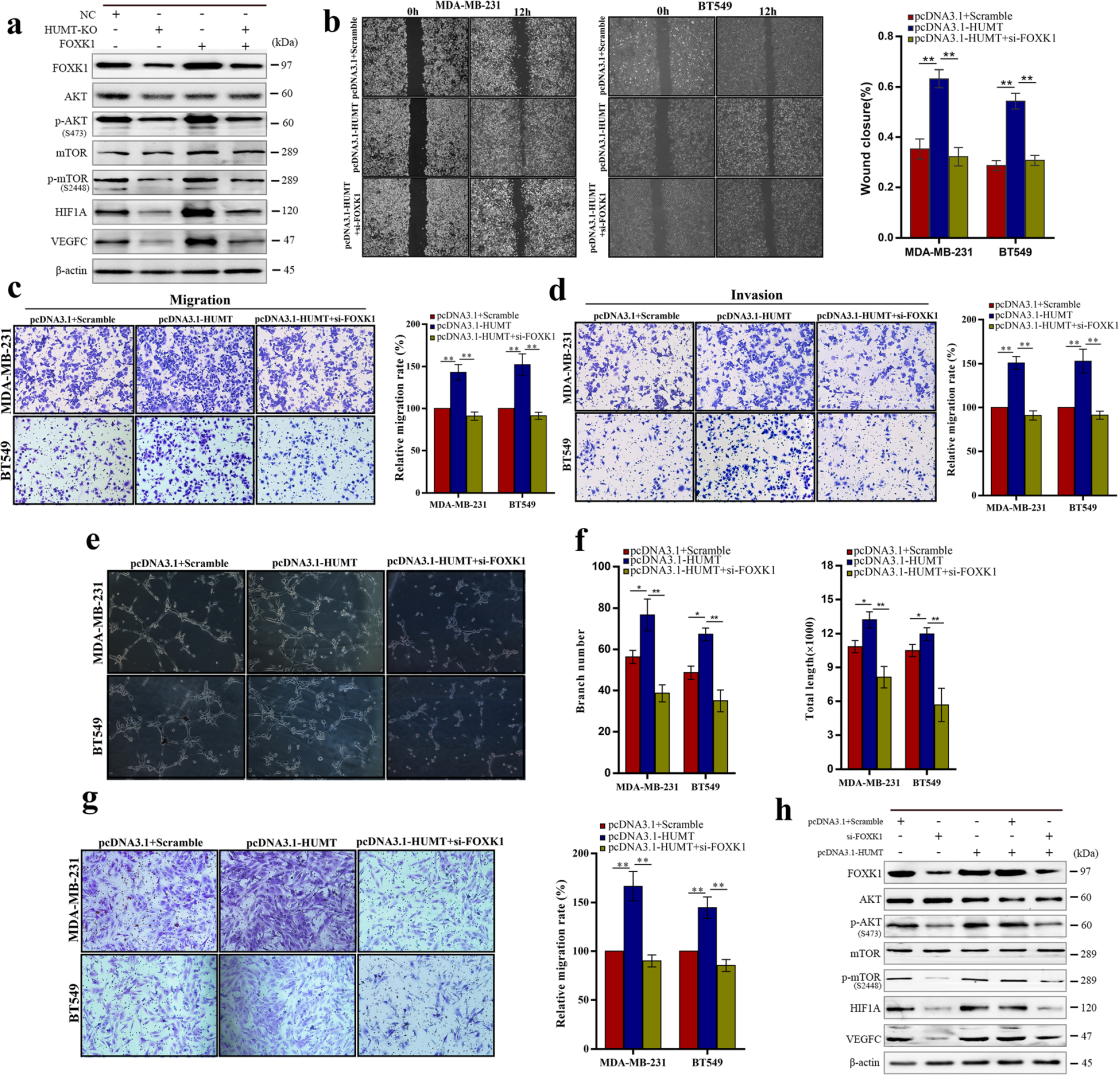
HUMT exerted its function by regulating the FOXK1 expression and downstream signaling. **a** Western blot analysis of the corresponding signaling in HUMT-KO- and FOXK1-overexpressed MDA-MB-231 cells. **b**–**d** Representative graphs and quantification of wound healing assay, Transwell migration, and invasion assay in the MDA-MB-231 and BT549 cells cotransfected with HUMT overexpression vector or empty vector together with si-FOXK1 or scrambled control. **e** Representative pictures of tube formation assay in HLECs cultured in medium supernatant of the abovementioned cells. **f** Quantitative analysis of the branch number and total tube length in tube formation assay. **g** Representative graphs and quantification of Transwell migration assay in HLECs cultured in medium supernatant of the abovementioned cells. **h** Western blot analysis of the corresponding signaling in MDA-MB-231 and BT549 cotransfected with HUMT overexpression vector or empty vector together with si-FOXK1 or scrambled control. Data were shown as mean ± SD; *P < 0.05; **P < 0.01

Previous studies have identified a crucial role of FOXK1 in cancers [33–35]. To explore whether HUMT exerted its function through FOXK1-mediated cell proliferation and vascular endothelial growth factor (VEGFC) signaling, which was a previously reported key lymphangiogenesis pathway [36, 37], we analyzed the effect of HUMT-KO by western blot. Our results showed that the protein level of FOXK1, p-Akt, p-mTOR, and VEGFC was markedly downregulated in response to HUMT-KO, but it could be partially reversed by FOXK1 overexpression in MDA-MB-231 cells (Fig. 5a).

To further validate the role of FOXK1 in HUMT-mediated tumor progression, we transfected MDA-MB-231 and BT549 cells stably overexpressing HUMT or empty vector with si-FOXK1 or scramble vector. An in vitro experiment showed that knockdown of FOXK1 could reverse the effect of HUMT overexpression. The effect of promoting migration and invasion by HUMT overexpression was abolished by the FOXK1 knockdown (Fig. 5b–d). The tube formation assay showed that the effect of promoting lymph node metastasis by HUMT overexpression could be reversed by the FOXK1 knockdown (Fig. 5e, f). Moreover, the migration activity of HLECs was also affected in the Transwell assay (Fig. 5g). Western blot of the rescue experiments showed that FOXK1 knockdown could partially reverse the increased expression of key signalings mediated by HUMT in MDA-MB-231 cells (Fig. 5h).

Taken together, our findings indicated that lncRNA HUMT promotes cancer cell proliferation, lymph node metastasis, and lymphangiogenesis by HUMT/YBX1/FOXK1-mediated Akt/mTOR and VEGFC signaling.

### HUMT promotes triple-negative breast cancer cell proliferation, metastasis, and lymphangiogenesis in vivo

**Fig. 6:**
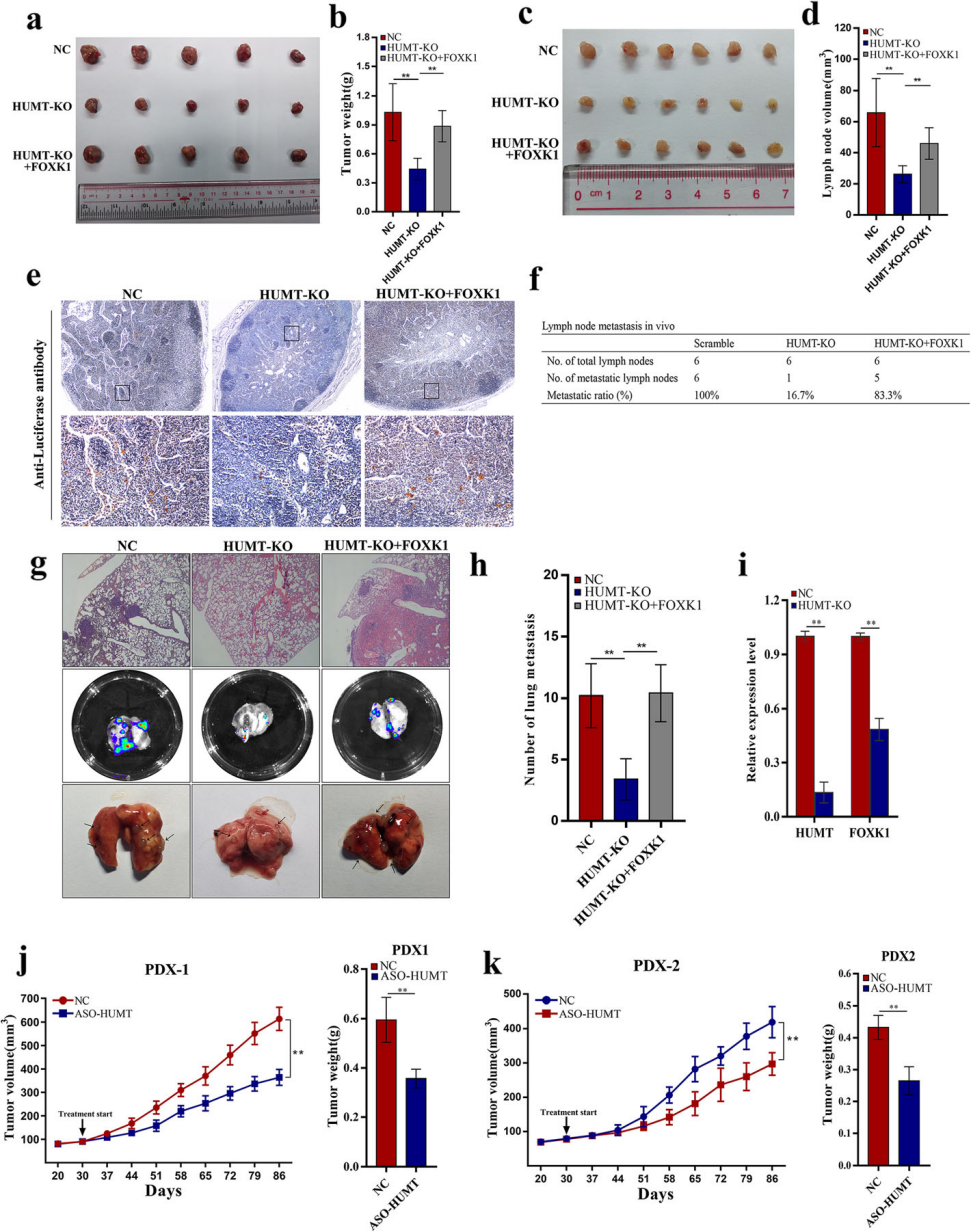
HUMT promotes cell proliferation and metastasis in vivo. **a** Representative picture of the subcutaneous graft model in nude mice using corresponding modulated 231LNM3 cells. **b** Tumor weight after implantation of corresponding cells. **c**, **d** The representative picture (231LNM3) and the corresponding volume of lymph nodes in different groups. **e** Representative picture of immunohistochemical staining of lymph nodes in different groups. **f** The metastatic ratio of lymph nodes in different groups. **g** Representative picture of metastatic mice lung, bioluminescence, and HE staining in different groups. **h** Quantification of metastatic lung nodules. **i** HUMT and FOXK1 expression in tumors were examined by qRT-PCR. **j**, **k** Tumor growth volume and tumor weights in two PDX models of intratumoral treatment with ASO-HUMT and NC. Data were shown as mean ± SD; **P < 0.01

We further conducted experiments in vivo to validate the oncogenic characteristics of HUMT and FOXK1. The xenograft model showed that HUMT-KO could significantly suppress tumor growth compared with the control group, and it could be reversed by FOXK1 overexpression (Fig. 6a, b). Lymph node metastasis was widely considered as the first step for breast cancer metastasis, and cancer cells further extended to distant sites, especially lung metastasis [38]. To confirm the function of HUMT in triple-negative breast cancer metastasis in vivo, the lymph node metastasis and lung metastasis model of 231LNM3 were established. The mice in HUMT-KO exhibited a lower volume of lymph nodes compared with controls, and it could be reversed by FOXK1 overexpression (Fig 6c, d). The lymph nodes of nude mice were removed for IHC stain, and metastatic cancer cells were significantly less enriched in the HUMT-KO group than controls (Fig. 6e). Besides, the ratio of metastatic lymph nodes was significantly decreased in the HUMT-KO group (Fig. 6f). HUMT-KO could also suppress lung metastasis in nude mice (Fig. 6g, h). We further confirmed in the tumor that HUMT-KO significantly downregulated FOXK1 expression by qRT-PCR (Fig. 6i).

As patient-derived xenograft (PDX) models reflect the effect of microenvironment on tumor growth and treatment response for the origin patients, we further explored the potential role of HUMT as a therapeutic target by intratumoral ASO injection. The tumor volumes and tumor weights of the HUMT-KD group were significantly lower compared with the control group (Fig. 6j, k). These results revealed that HUMT-KO significantly inhibited tumor proliferation in vivo.

Taken together, HUMT promotes tumor growth and metastasis in vivo.

### The clinical relevance of HUMT in patients with TNBC

**Fig. 7:**
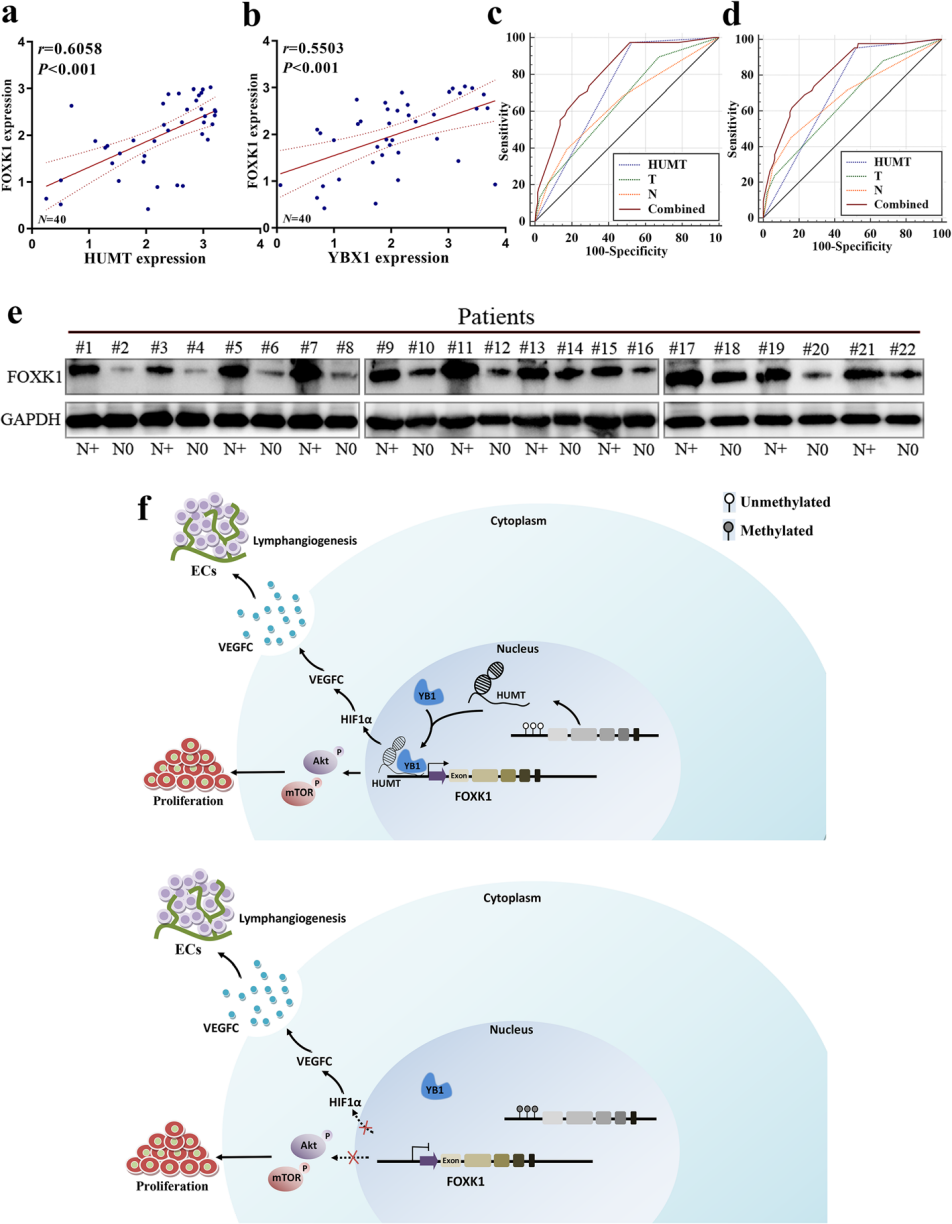
Clinical relevance and schematic mechanism of HUMT in TNBC. **a**, **b** Correlation between HUMT, YBX1, and FOXK1 in clinical TNBC samples of SYSUCC (*N* = 40). **c** ROC analysis of OS for AJCC T stage [AUC = 0.651 (95%CI, 0.558–0.743)], N stage [AUC = 0.641 (95%CI, 0.540–0.742)], HUMT [AUC = 0.724 (95%CI, 0.652–0.795)], and combined panel [AUC = 0.808 (95%CI, 0.739–0.877)]. **d** ROC analysis of DFS for AJCC T stage [AUC = 0.658 (95%CI, 0.567–0.749)], N stage [AUC = 0.683 (95%CI, 0.587–0.780)], HUMT [AUC = 0.715 (95%CI, 0.643–0.788)], and combined panel [AUC = 0.816 (95%CI, 0.750–0.882)]. **e** WB analysis showed a higher level of FOXK1 in patients with lymph node metastatic TNBC. **f** The proposed mechanism of HUMT in this study

We used the KM plotter (http://kmplot.com/analysis/) to evaluate the prognostic value of HUMT in different cancers. HUMT was correlated with poorer OS and relapse-free survival (RFS) in specific cancers, not just in basal-like breast cancer (Additional file 7: Fig. S7). Moreover, the clinical relevance of HUMT and FOXK1 was assessed in patients with TNBC from SYSUCC. The qRT-PCR analysis confirmed the significant correlations between HUMT and YBX1, FOXK1, which was consistent with the results above (Fig. 7a, b). ROC analysis of OS and DFS revealed that a combined panel of HUMT expression, T stage, and N stage showed an additional predictive value (Fig. 7c, d). Western blot showed a higher level of FOXK1 protein in triple-negative breast cancer patients with metastatic lymph nodes (Fig. 7e), further confirming a crucial role in lymph node metastasis.

In summary, we put forward a proposed mechanism for HUMT-mediated triple-negative breast cancer progression, including cell proliferation and lymph node metastasis (Fig. 7f). Interestingly, the bioinformatic analysis indicated that HUMT upregulation was correlated with a lower fraction of activated NK cells calculated by CIBERSORT in TNBC from TCGA and GSE58812 (Additional file 8: Fig. S8a). The NK cells were stained, and stromal NK cells were quantified. Patients with high FOXK1 expression in the TCGA-TNBC cohort also showed a higher level of TGF-β, which was considered as an immunosuppressive factor in cancer (Additional file 8: Fig. S8b). We found that patients with high-HUMT expression exhibited a lower rate of positive stromal NK cells (Additional file 8: Fig. S8c, d). Moreover, HUMT might be correlated with cancer cell stemness as fewer spheroids were observed in the HUMT-KO group compared with the controls, indicating that the repression of HUMT could inhibit the stemness feature of MDA-MB-231 and BT549 (Additional file 9: Fig. S9).

Considering that FOXK1 was reported to be associated with M2-type macrophages attraction and thus promoted tumor growth [39], the HUMT/YBX1/FOXK1 axis might also be functioning in immune escape. But the mechanism of these results still needs further exploration in the future.

## Discussions

Axillary lymph nodes were usually the first step for breast cancer metastasis. Identification of novel biomarkers and molecular mechanisms for lymph node metastasis might help to stratify patients with different prognostic risks and provide effective treatment for patients with breast cancer. Triple-negative breast cancer is the most malignant subtype of breast cancer, which was easier to develop lymph node metastasis and has very limited therapeutic options at present. However, the question why lymph node metastasis was generally preferable was usually neglected. Hence, it is urgent to investigate the biological basis of cancer proliferation and lymph node metastasis and further identify novel targets for TNBC treatment.

In this study, we firstly identified a novel lncRNA HUMT that was highly upregulated in lymph node-invasive cells and explore the corresponding mechanism. The results showed that HUMT was significantly upregulated due to DNA hypomethylation at the promoter region and promoted tumor proliferation and lymph node metastasis. Further study indicated that HUMT exerted its function by recruiting YBX1 protein to the promoter region of FOXK1 and promoted its transcription, activating lymph node metastasis-related Akt/mTOR/VEGFC signaling. Besides, tumor proliferation was generally tightly involved in the process of metastasis [27, 30, 40]. Our study confirmed the important role of HUMT in cancer cell proliferation in vivo and in vitro, which was consistent with the natural process.

Previous studies have addressed the crucial functions of lncRNA involved in tumor proliferation and metastasis. LncRNAs could serve as potential biomarkers and therapeutic targets in cancers. However, many studies mainly focused on distant metastasis, such as the bone, lung, liver, and brain. The role of lncRNAs and their therapeutic values for triple-negative breast cancer lymph node metastasis still remain rarely studied. Besides, lncRNAs were reported to be involved in lymph node metastasis in other cancers. For example, LNMICC was an oncogenic lncRNA that could be inhibited by miR-190 and could promote lymph node metastasis via FABP5-mediated FA metabolism [40]. In our study, the prognostic value of HUMT was confirmed in a Sun Yat-sen University Cancer Center cohort of triple-negative breast cancer. Our data showed that HUMT was significantly upregulated in highly lymph node-invasive cells, and a higher level of HUMT was associated with poor clinical prognosis and lymph node metastasis in patients with TNBC. Functional study in vivo and in vitro showed that HUMT promoted lymphangiogenesis and metastasis. Hence, we have good reasons to suppose that HUMT creates a favorable condition for lymph node metastasis in TNBC.

Increasing studies have reported that lncRNA could promote the transcriptions by binding to specific regions at the promoter [29]. In our study, we demonstrated that HUMT recruited YBX1, a well-known RNA/DNA-binding protein and formed the transcription complex to trigger the expression of FOXK1, leading to downstream alteration. These results suggested that a novel HUMT/YBX1/FOXK1 axis played a crucial role in triple-negative breast cancer lymph node metastasis. Interestingly, since both YBX1 and FOXK1 served as transcription factors in the nuclei of cancer cells, overexpression of lncRNA HUMT might lead to a chain reaction that could amplify the functions of upstream signals. Our results also supported that the methylation status of the promoter region modulated the expression level of HUMT and further regulated the downstream signals underlying lymph node metastasis and even distant metastasis in TNBC.

Emerging evidence revealed that lncRNAs were associated with cancer microenvironment and immune response [41]. Previous studies have reported that tumor-infiltrating immune cells played an important role in breast cancer prognosis and metastasis [42, 43]. In this study, the function of HUMT was verified in PDX models with consistent physical microenvironment and internal heterogeneity of original patients. The bioinformatic analysis showed that a higher level of HUMT was correlated with a lower fraction of activated NK cells, which indicated that HUMT might lead to an immunosuppressive microenvironment and promote cell proliferation and metastasis. The result was verified by immunohistochemistry in cancer tissues. Further study confirmed this notion because FOXK1 was significantly positively correlated with TGF-β, which were well-known immunosuppressive factors. This interesting result is worth further exploration. Taken together, these findings might partially explain the functions of lncRNAs in lymph node metastasis of TNBC, but further biological mechanism underlying non-coding RNA still deserves extensive study.

However, there are still some limitations to this study. First, the specific mechanism of hypomethylation at the promoter region of HUMT was not clearly elucidated. Neither some DNA methylation-related factors, such as the DNMT family, nor the genome-wide methylation level was examined. The specific mechanism needs further exploration. Next, as about half of HUMT was located in the cytoplasm, competing endogenous RNA network or protein-stabilizing could be another mechanism of how HUMT exerted its function.

### Conclusions

In conclusion, our study has firstly proved a novel target, HUMT, for triple-negative breast cancer lymphangiogenesis and metastasis. Epigenetics and transcription regulation represents the biological mechanism of HUMT in modulating cell proliferation and lymph node metastasis. Moreover, PDX models indicated silencing HUMT expression might serve as a novel therapeutic strategy.

## Supplementary information

**Additional file 1: Figure S1.** (a, b) Location and length of open read frame (ORF) in HUMT predicted by ORFfinder. (c) PhyloCSF was used to predict the coding potential of HUMT. (d) CPAT predicted a low probability for protein-coding potential for HUMT.

**Additional file 2: Figure S2.** Bioinformatic analysis of TCGA datasets indicated significantly upregulated HUMT expression in TNBC and other specific cancers. *, *P*<0.05; **, *P*<0.01; ***, *P*<0.001.

**Additional file 3: Figure S3.** HUMT promoted cancer proliferation via FOXK1. (a, b) CCK8 assay of MDA-MB-231 and BT549 cells transfected with control or HUMT-KO vectors. Representative graphs (left) and quantification (right) of colony formation assay. (c, d) CCK8 assay in MDA-MB-231 and BT549 cotransfected with HUMT overexpression vector or empty vector together with si-FOXK1 or scrambled control. Representative graphs (left) and quantification (right) of colony formation assay. **, *P*<0.01; ***, *P*<0.001.

**Additional file 4: Figure S4.** Pan-cancer analysis of TCGA datasets indicated a significant correlation between specific methylation probe signal and HUMT expression.

**Additional file 5: Figure S5.** (a) The nuclear distribution of HUMT was predicted using lncATLAS tools. (b) Nuclear and cytoplasmic distribution of HUMT in two independent cancer cells was detected by qRT-PCR. (c) RNA FISH analysis showed the intracellular location of HUMT.

**Additional file 6: Figure S6.** (a-c) Bioinformatic analysis of YBX1 CHIP-seq in three independent cell lines of the ENCODE database indicates binding sites at promoter region. (d) Predicted YBX1-binding motif on FOXK1 promoter region in three cell lines.

**Additional file 7:.** Figure S7. Kaplan-Meier analysis showed HUMT predicted a poorer OS and RFS outcomes in specific cancers using KM plotter tools.

**Additional file 8: Figure S8.** (a) CIBERSORT analysis of triple-negative breast cancer in GSE58812 and TCGA database showed the HUMT-high group presented with a higher level of activated NK cells. (b) In TNBC of the TCGA database, FOXK1 predicted a higher level of TGF-β1 and TGF-β2 expression. (c) NK cells in tumor core and stromal tissues of TNBC were stained. (d) HUMT-high status predicted a lower rate of positive stromal NK cells-infiltrating tumor microenvironment in TNBC. *, *P*<0.05; **, *P*<0.01.

**Additional file 9: Figure S9.** The tumor spheroids formed by cells in the HUMT-KO or control groups.

**Additional file 10:** Supplementary Material and Methods.

**Additional file 11: Table S1.**

**Additional file 12: Table S2.**

**Additional file 13: Table S3.**

**Additional file 14: Table S4.**

**Additional file 15: Table S5.**

**Additional file 16: Table S6.**

## Data Availability

All data generated or analyzed during this study are included in this published article. The datasets supporting the conclusions of this article are included within the article and its additional files.

## Abbreviations

CCLE: Cancer Cell Line Encyclopedia
CHIP: Chromatin immunoprecipitation
CPAT: Coding potential assessment tool
DAB: 3,3′-Diaminobenzidine
dCas9-CHIP: dCas9-gRNA-guided chromatin immunoprecipitation
DFS: Disease-free survival
FISH: Fluorescent in situ hybridization
FOXK1: Forkhead box k1
HE: Hematoxylin-eosin
HUMT: lncRNA highly upregulated in metastatic TNBC
IHC: Immunohistochemistry
ISH: In situ hybridization
KO: Knock-out
lncRNA: Long non-coding RNAs
LNMFS: Lymph node metastasis-free survival
MSP: Methylation-specific polymerase chain reaction
NC: Normal control
ORF: Open reading frame
OS: Overall survival
qRT-PCR: Quantitative real-time polymerase chain reaction
RFS: Relapse-free survival
RIP: RNA immunoprecipitation
ROC: Receiver operating characteristic
SYSUCC: Yat-sen University Cancer Center
TCGA: The Cancer Genome Atlas
TNBC: Triple-negative breast cancer
VEGFC: Vascular endothelial growth factor C
YBX1: Y-box binding protein 1
