# Correction to: Long non-coding RNA HUMT hypomethylation promotes lymphangiogenesis and metastasis via activating FOXK1 transcription in triple-negative breast cancer

**DOI:** 10.1186/s13045-020-00861-x

**Published:** 2020-03-24

**Authors:** Shaoquan Zheng, Lu Yang, Yutian Zou, Jie-ying Liang, Peng Liu, Guanfeng Gao, Anli Yang, Hailin Tang, Xiaoming Xie

**Affiliations:** 1 Department of Breast Oncology, Sun Yat-sen University Cancer Center, 651 East Dongfeng Road, Guangzhou, 510060 China; 2 Department of Medical Oncology, Sun Yat-sen University Cancer Center, 651 East Dongfeng Road, Guangzhou, 510060 China; 3 State Key Laboratory of Oncology in South China, Collaborative Innovation Center for Cancer Medicine, Sun Yat-sen University Cancer Center, 651 East Dongfeng Road, Guangzhou, 510060 Guangdong China

**Correction to: J Hematol Oncol**

**https://doi.org/10.1186/s13045-020-00852-y**

The original article [1] contains an error in Fig. 5b whereby two panels have been mistakenly duplicated. The correct version of Fig. 5b can be viewed ahead alongside the rest of Fig. 5.

**Fig. 5:**
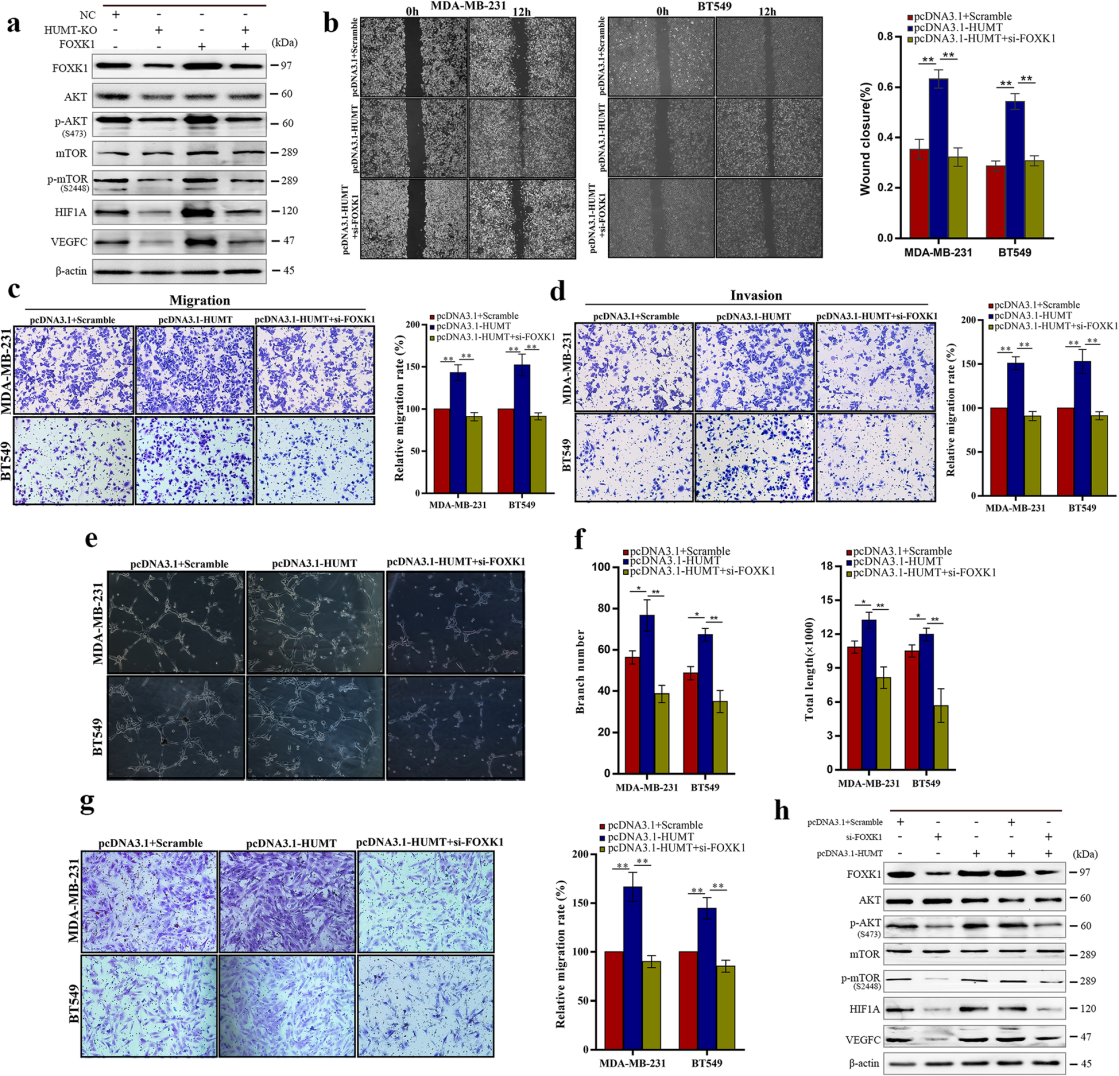
HUMT exerted its function by regulating the FOXK1 expression and downstream signaling. **a** Western blot analysis of the corresponding signaling in HUMT-KO- and FOXK1-overexpressed MDA-MB-231 cells. **b**–**d** Representative graphs and quantification of wound healing assay, Transwell migration, and invasion assay in the MDA-MB-231 and BT549 cells cotransfected with HUMT overexpression vector or empty vector together with si-FOXK1 or scrambled control. **e** Representative pictures of tube formation assay in HLECs cultured in medium supernatant of the abovementioned cells. **f** Quantitative analysis of the branch number and total tube length in tube formation assay. **g** Representative graphs and quantification of Transwell migration assay in HLECs cultured in medium supernatant of the abovementioned cells. **h** Western blot analysis of the corresponding signaling in MDA-MB-231 and BT549 cotransfected with HUMT overexpression vector or empty vector together with si-FOXK1 or scrambled control. Data were shown as mean ± SD; *P < 0.05; **P < 0.01
