# Supplementary figures and images for: Long non-coding RNA HUMT hypomethylation promotes lymphangiogenesis and metastasis via activating FOXK1 transcription in triple-negative breast cancer
Source: J Hematol Oncol. 2020 Mar 5;13:17. doi: 10.1186/s13045-020-00852-y (PMC7059688; doi:10.1186/s13045-020-00852-y)

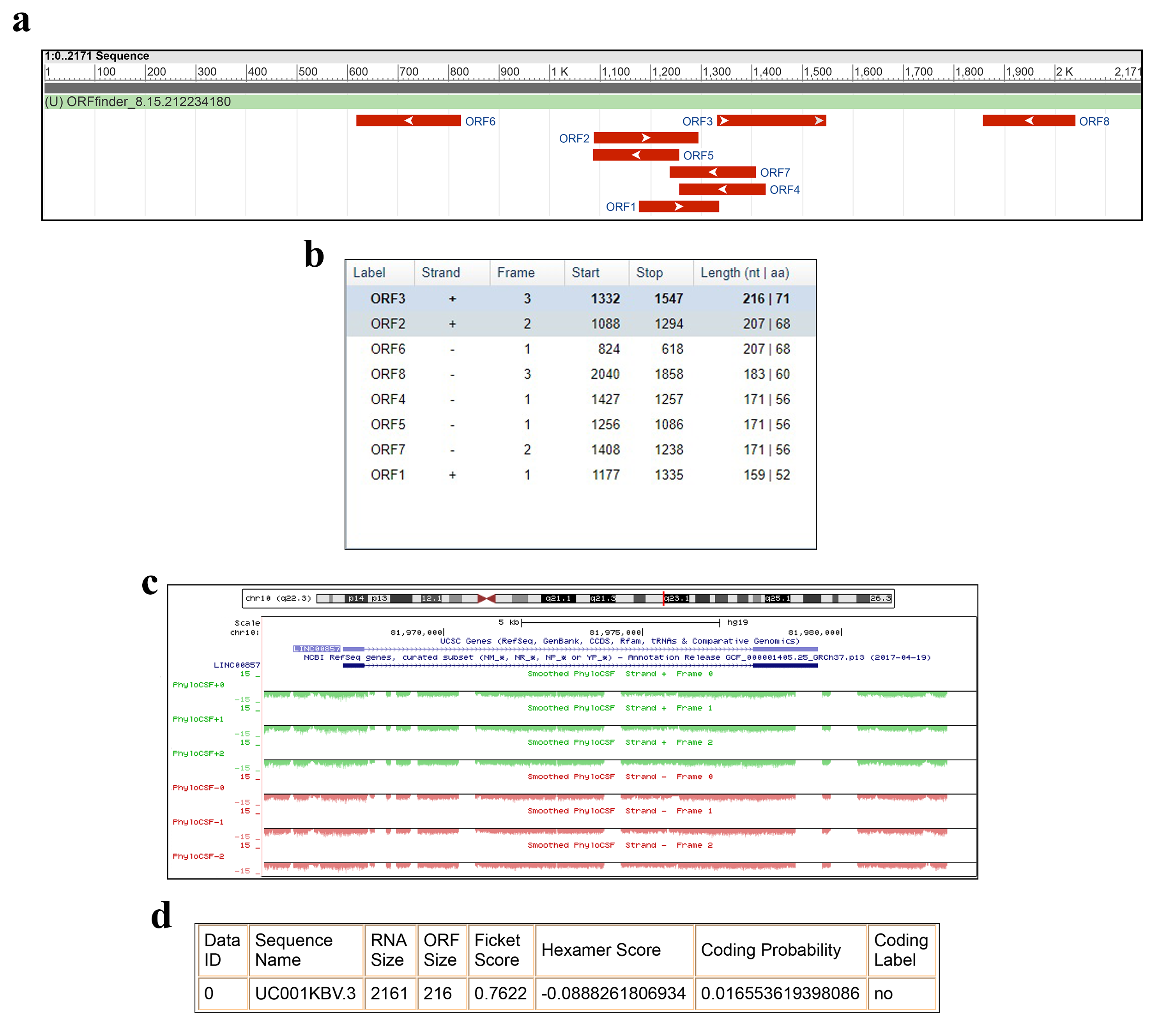

Supplement: Supplementary file 1 — Additional file 1: Figure S1. (a, b) Location and length of open read frame (ORF) in HUMT predicted by ORFfinder. (c) PhyloCSF was used to predict the coding potential of HUMT. (d) CPAT predicted a low probability for protein-coding potential for HUMT. [file 13045_2020_852_MOESM1_ESM.tif]

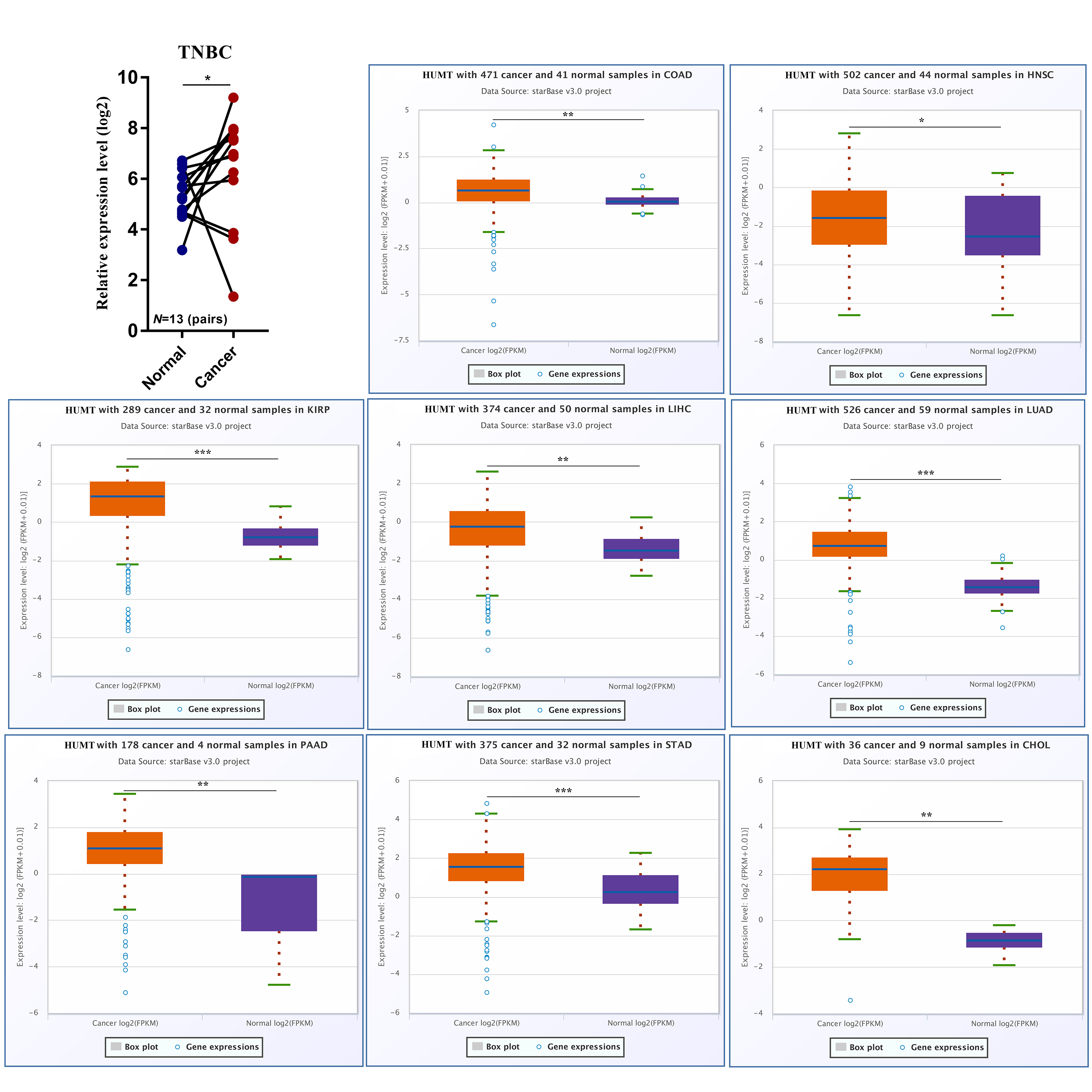

Supplement: Supplementary file 2 — Additional file 2: Figure S2. Bioinformatic analysis of TCGA datasets indicated significantly upregulated HUMT expression in TNBC and other specific cancers. *, P<0.05; **, P<0.01; ***, P<0.001. [file 13045_2020_852_MOESM2_ESM.tif]

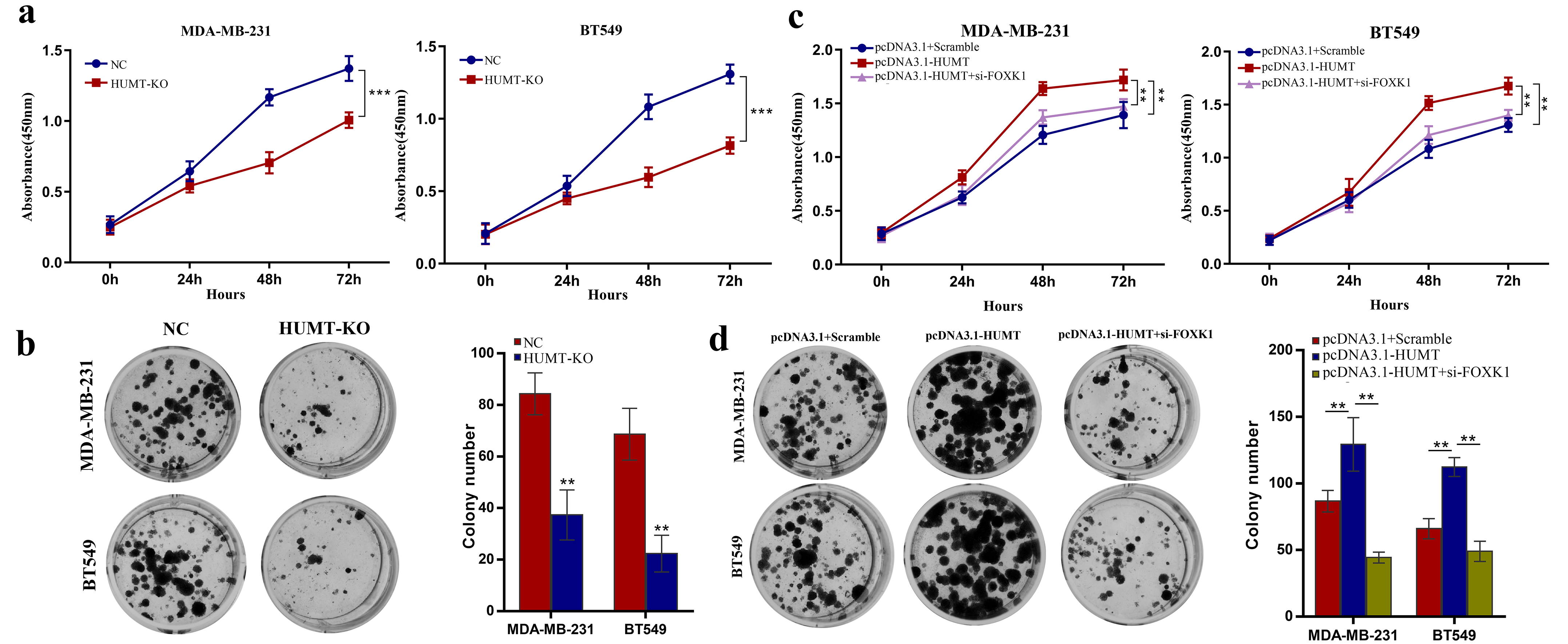

Supplement: Supplementary file 3 — Additional file 3: Figure S3. HUMT promoted cancer proliferation via FOXK1. (a, b) CCK8 assay of MDA-MB-231 and BT549 cells transfected with control or HUMT-KO vectors. Representative graphs (left) and quantification (right) of colony formation assay. (c, d) CCK8 assay in MDA-MB-231 and BT549 cotransfected with HUMT overexpression vector or empty vector together with si-FOXK1 or scrambled control. Representative graphs (left) and quantification (right) of colony formation assay. **, P<0.01; ***, P<0.001. [file 13045_2020_852_MOESM3_ESM.tif]

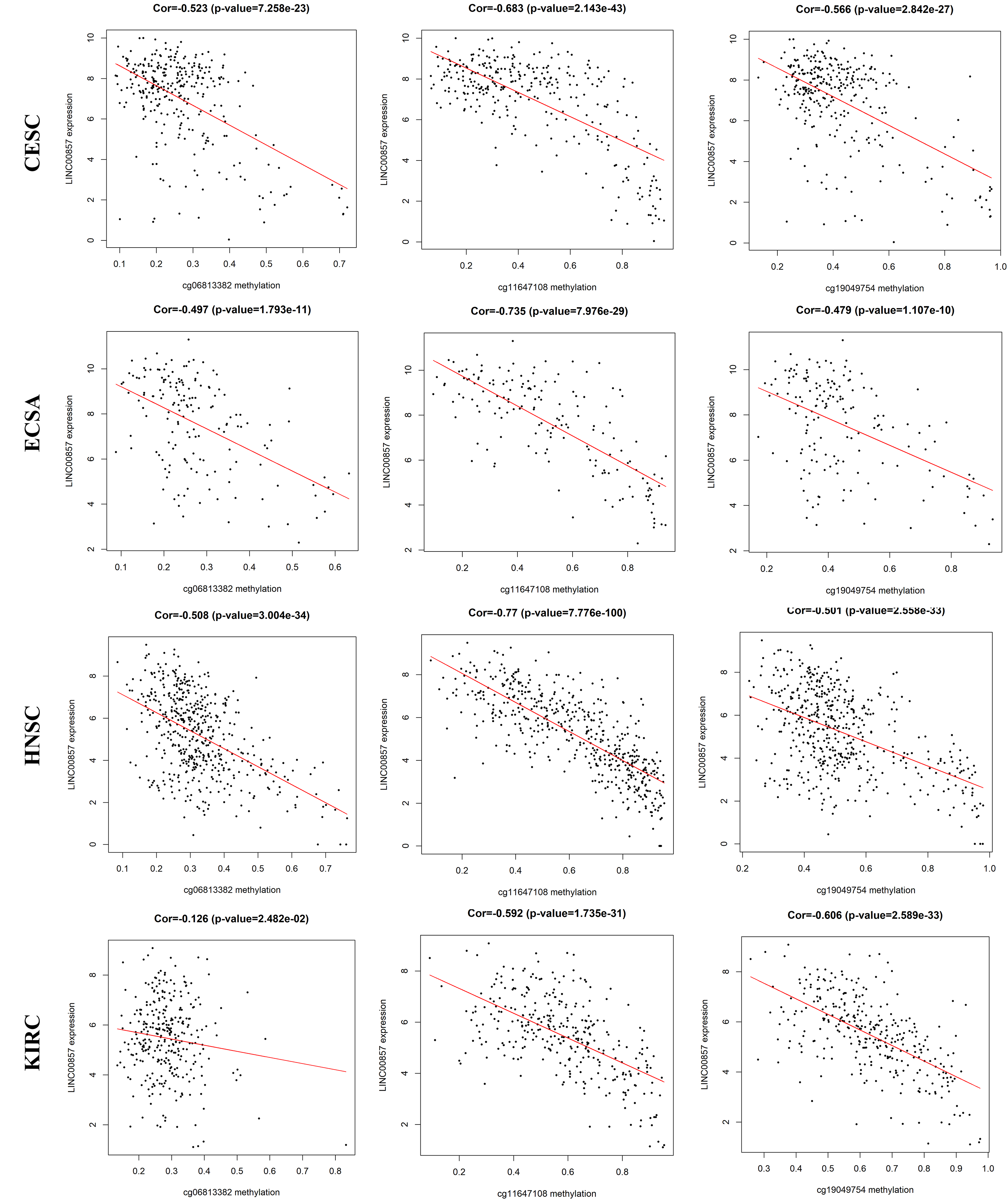

Supplement: Supplementary file 4 — Additional file 4: Figure S4. Pan-cancer analysis of TCGA datasets indicated a significant correlation between specific methylation probe signal and HUMT expression. [file 13045_2020_852_MOESM4_ESM.zip › Fig S4-1.tif]

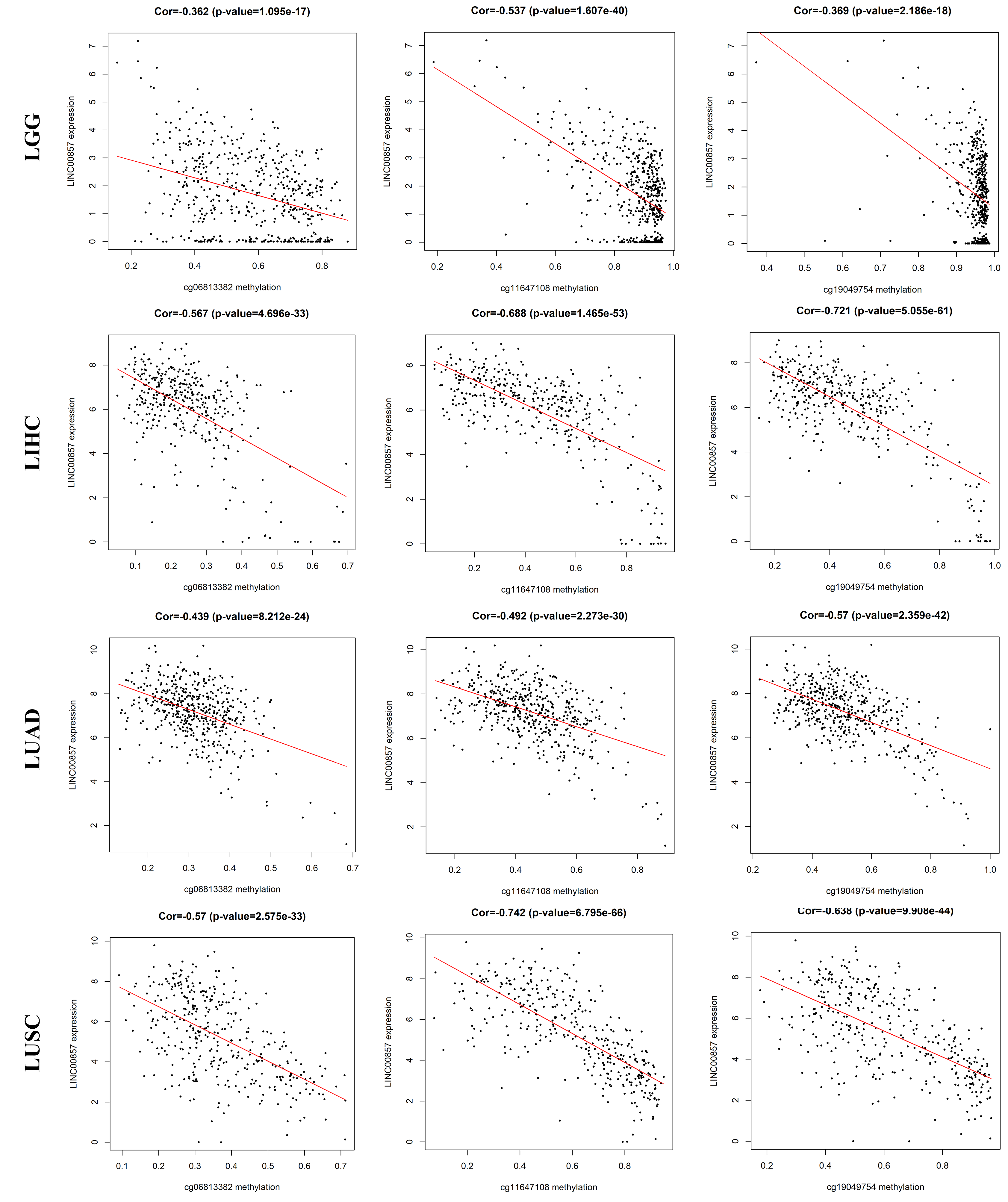

Supplement: Supplementary file 4 — Additional file 4: Figure S4. Pan-cancer analysis of TCGA datasets indicated a significant correlation between specific methylation probe signal and HUMT expression. [file 13045_2020_852_MOESM4_ESM.zip › Fig S4-2.tif]

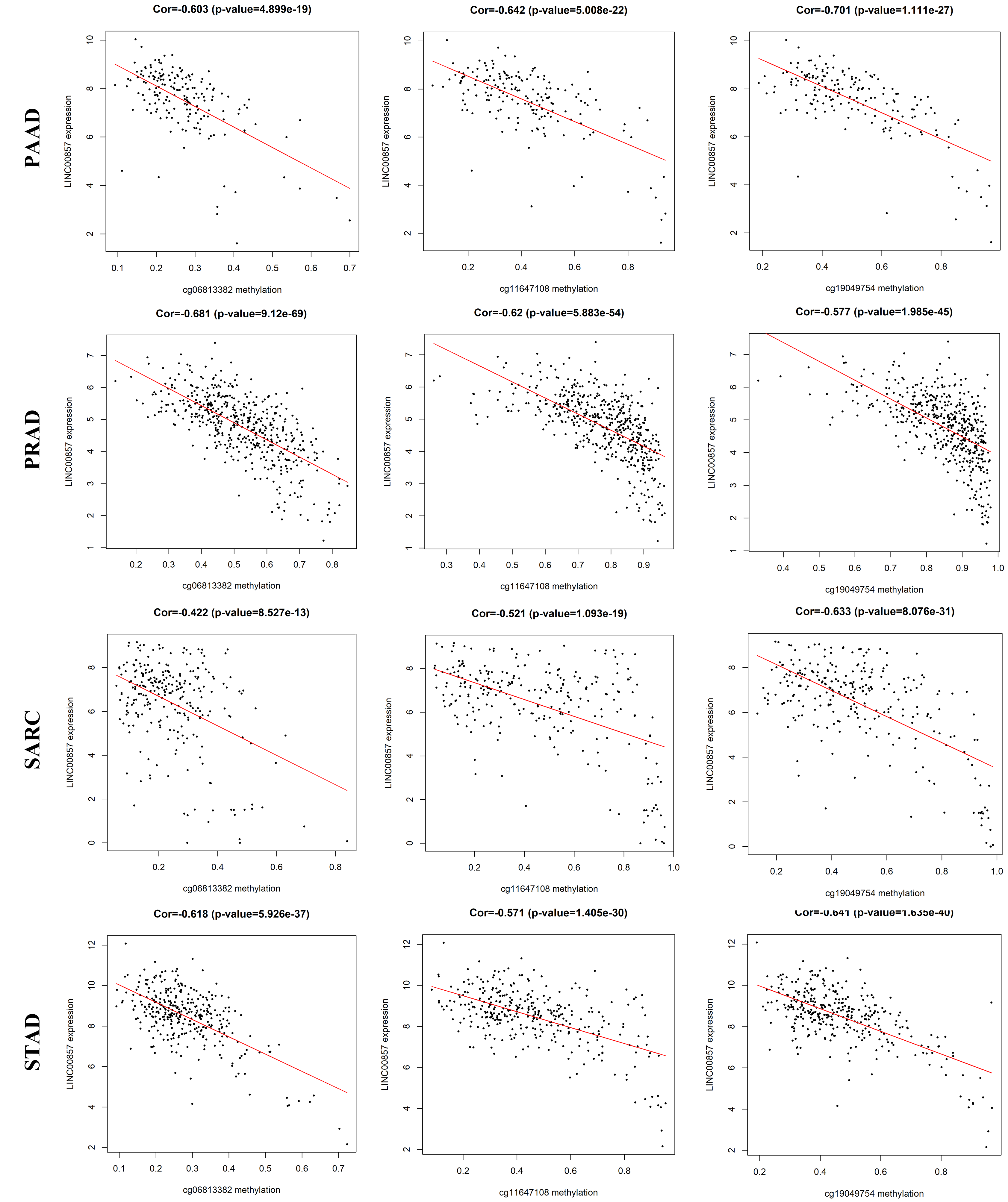

Supplement: Supplementary file 4 — Additional file 4: Figure S4. Pan-cancer analysis of TCGA datasets indicated a significant correlation between specific methylation probe signal and HUMT expression. [file 13045_2020_852_MOESM4_ESM.zip › Fig S4-3.tif]

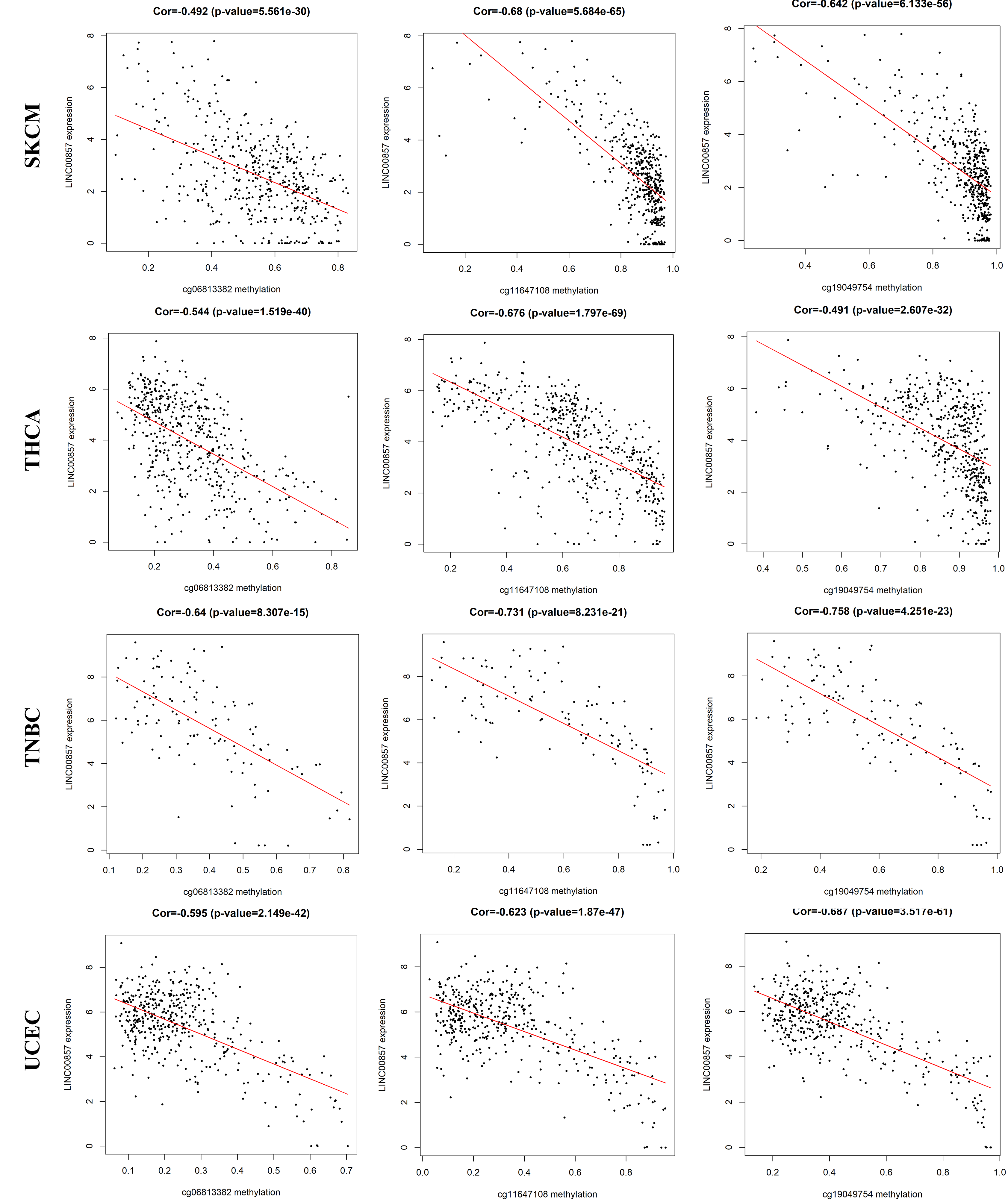

Supplement: Supplementary file 4 — Additional file 4: Figure S4. Pan-cancer analysis of TCGA datasets indicated a significant correlation between specific methylation probe signal and HUMT expression. [file 13045_2020_852_MOESM4_ESM.zip › Fig S4-4.tif]

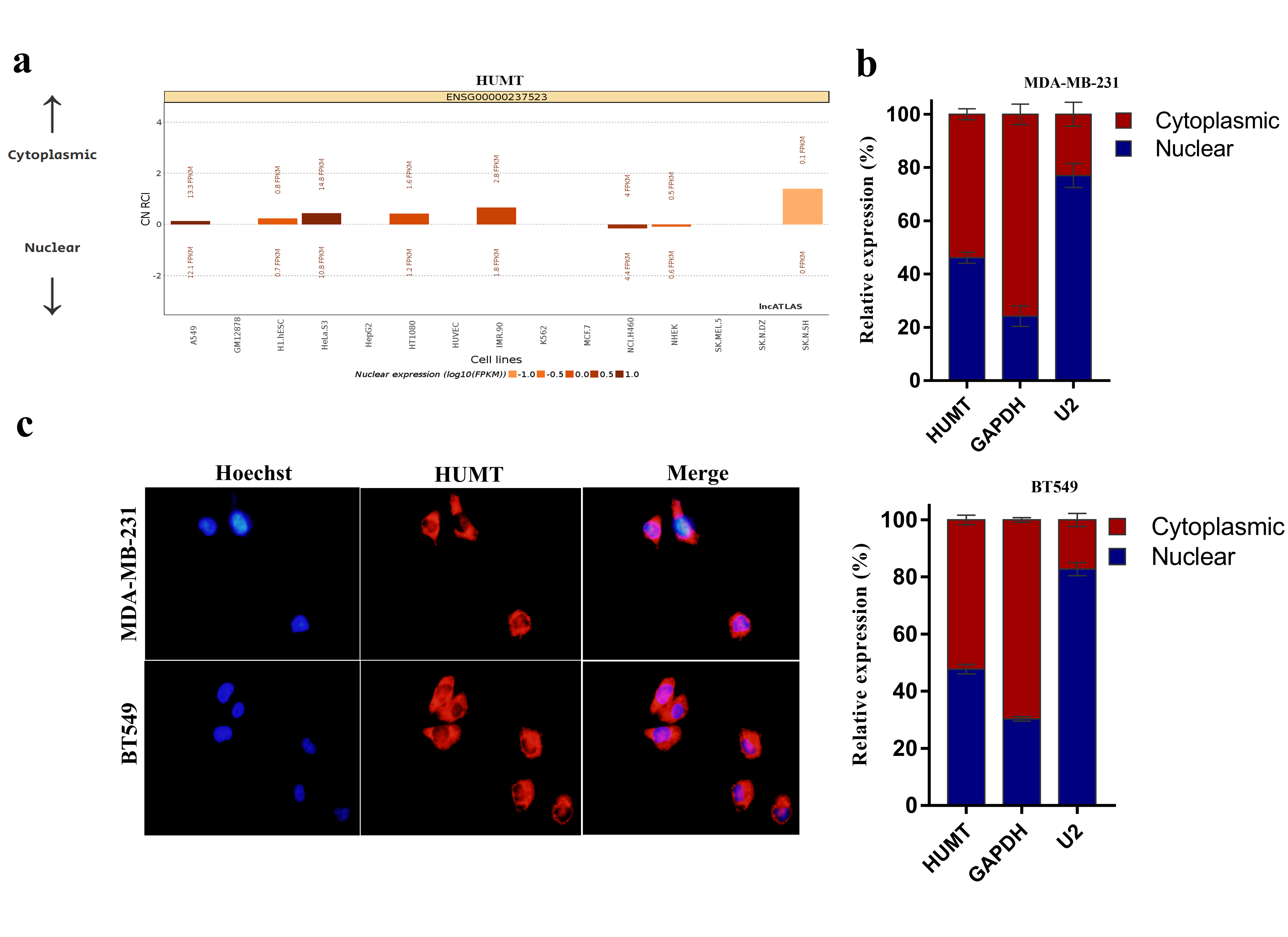

Supplement: Supplementary file 5 — Additional file 5: Figure S5. (a) The nuclear distribution of HUMT was predicted using lncATLAS tools. (b) Nuclear and cytoplasmic distribution of HUMT in two independent cancer cells was detected by qRT-PCR. (c) RNA FISH analysis showed the intracellular location of HUMT. [file 13045_2020_852_MOESM5_ESM.tif]

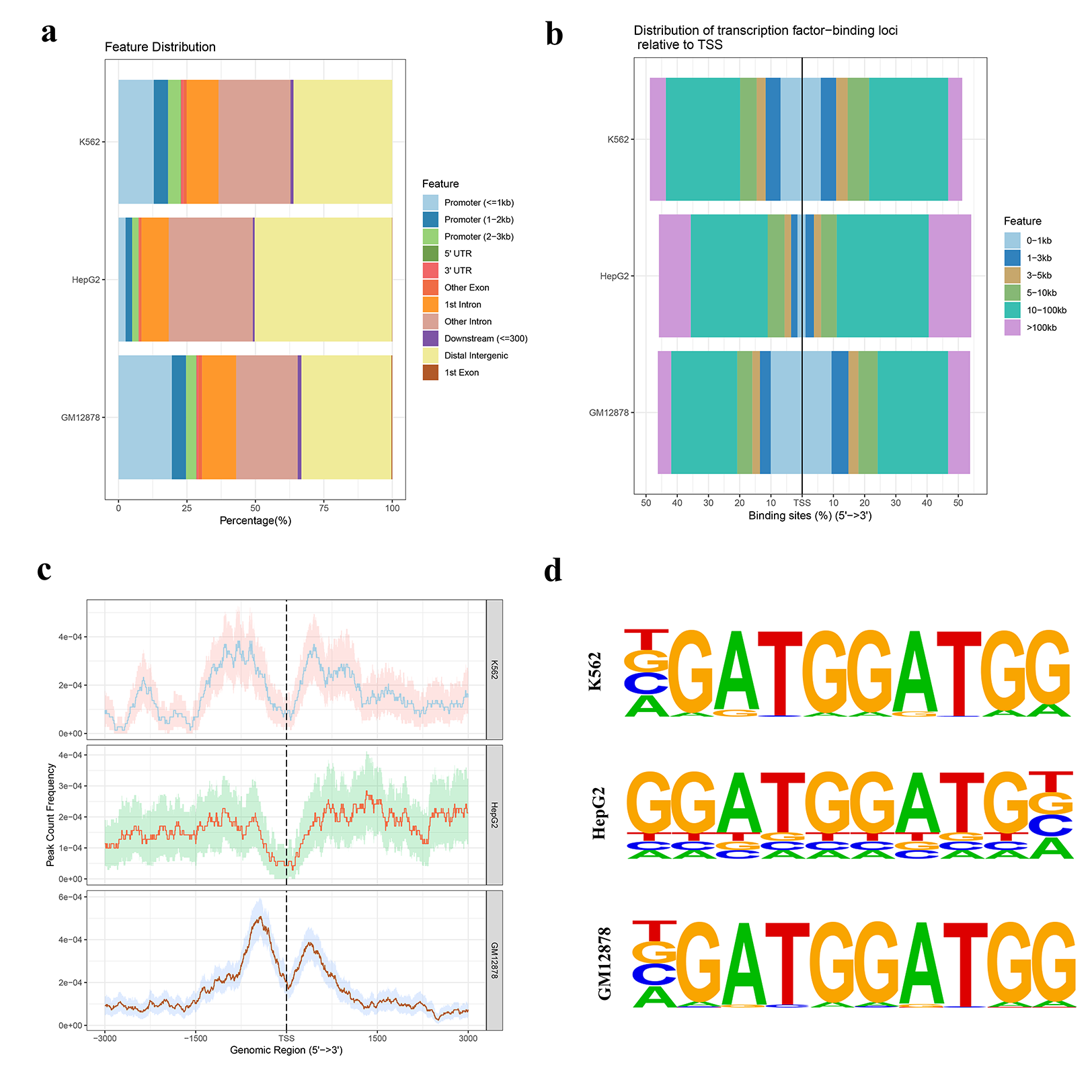

Supplement: Supplementary file 6 — Additional file 6: Figure S6. (a-c) Bioinformatic analysis of YBX1 CHIP-seq in three independent cell lines of the ENCODE database indicates binding sites at promoter region. (d) Predicted YBX1-binding motif on FOXK1 promoter region in three cell lines. [file 13045_2020_852_MOESM6_ESM.tif]

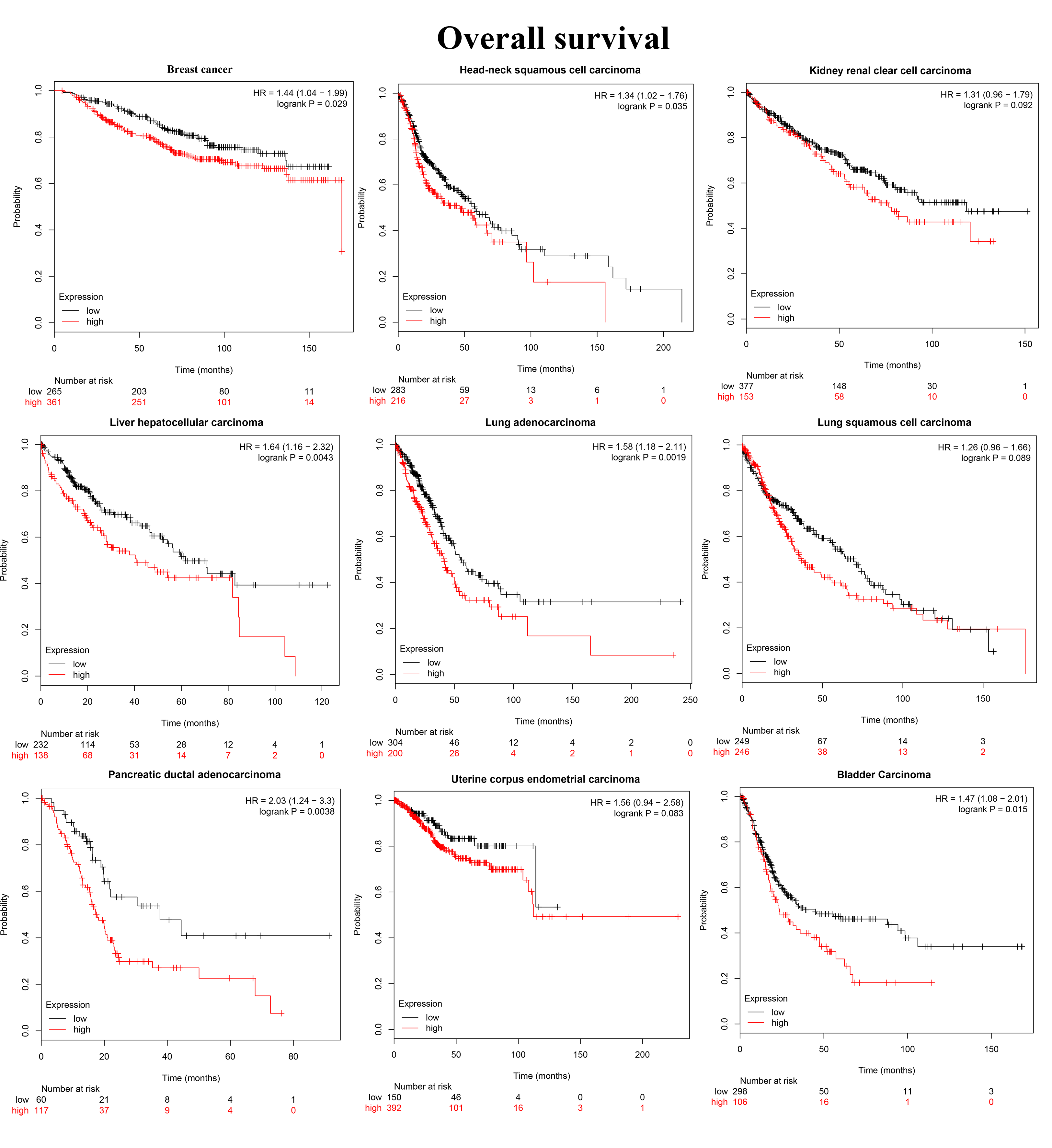

Supplement: Supplementary file 7 — Additional file 7:. Figure S7. Kaplan-Meier analysis showed HUMT predicted a poorer OS and RFS outcomes in specific cancers using KM plotter tools. [file 13045_2020_852_MOESM7_ESM.zip › Fig S7-1.tif]

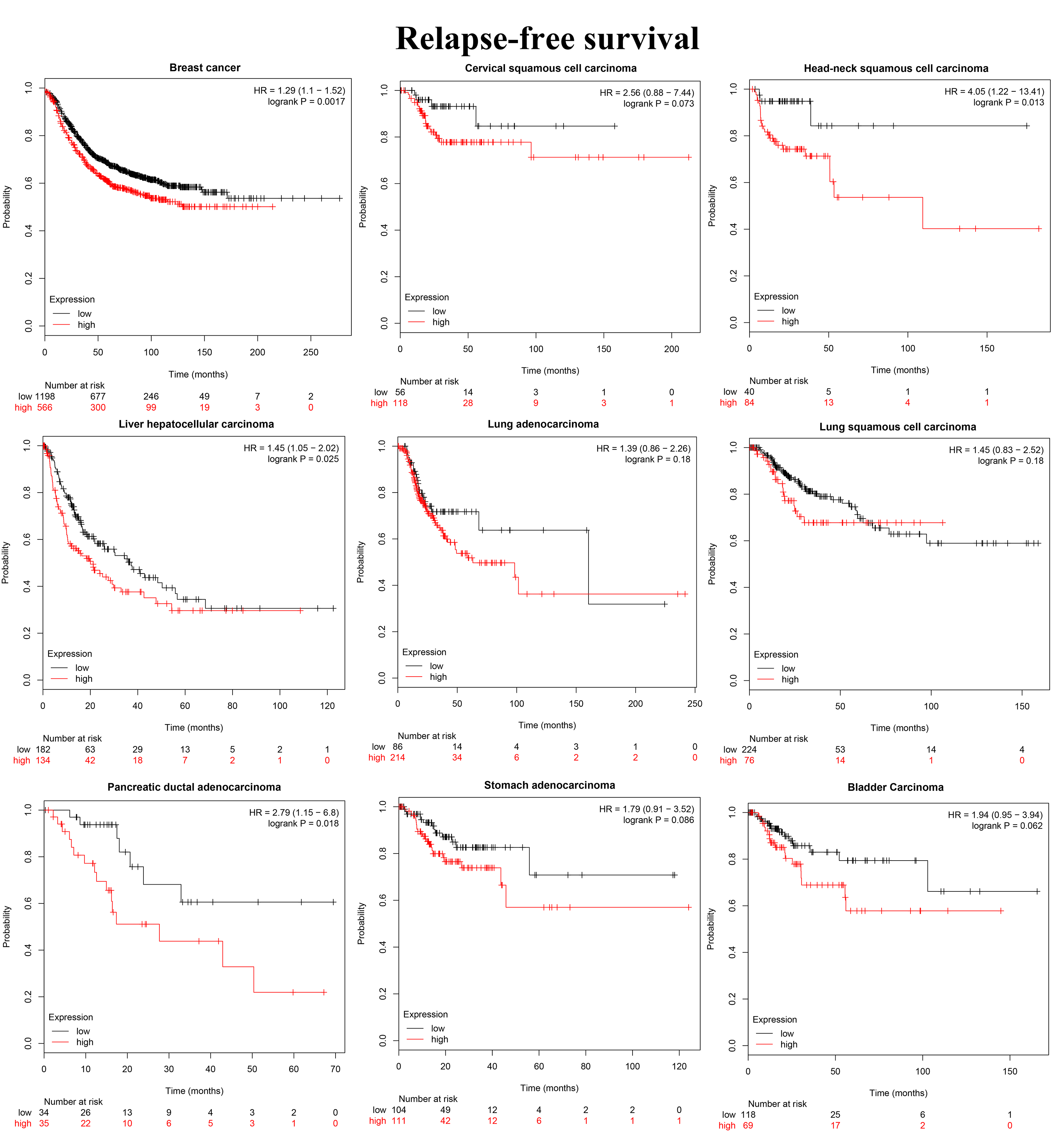

Supplement: Supplementary file 7 — Additional file 7:. Figure S7. Kaplan-Meier analysis showed HUMT predicted a poorer OS and RFS outcomes in specific cancers using KM plotter tools. [file 13045_2020_852_MOESM7_ESM.zip › Fig S7-2.tif]

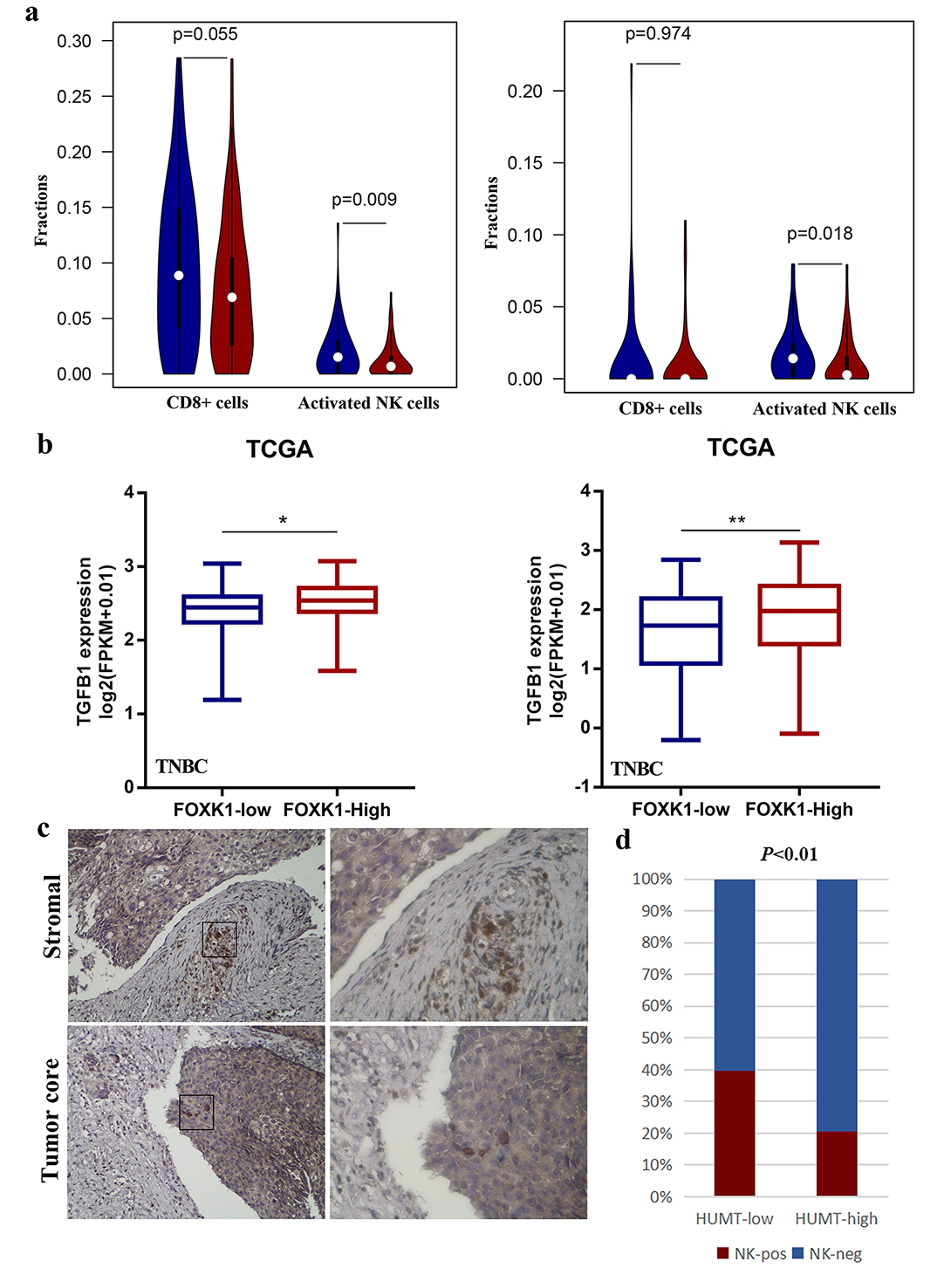

Supplement: Supplementary file 8 — Additional file 8: Figure S8. (a) CIBERSORT analysis of triple-negative breast cancer in GSE58812 and TCGA database showed the HUMT-high group presented with a higher level of activated NK cells. (b) In TNBC of the TCGA database, FOXK1 predicted a higher level of TGF-β1 and TGF-β2 expression. (c) NK cells in tumor core and stromal tissues of TNBC were stained. (d) HUMT-high status predicted a lower rate of positive stromal NK cells-infiltrating tumor microenvironment in TNBC. *, P<0.05; **, P<0.01. [file 13045_2020_852_MOESM8_ESM.tif]

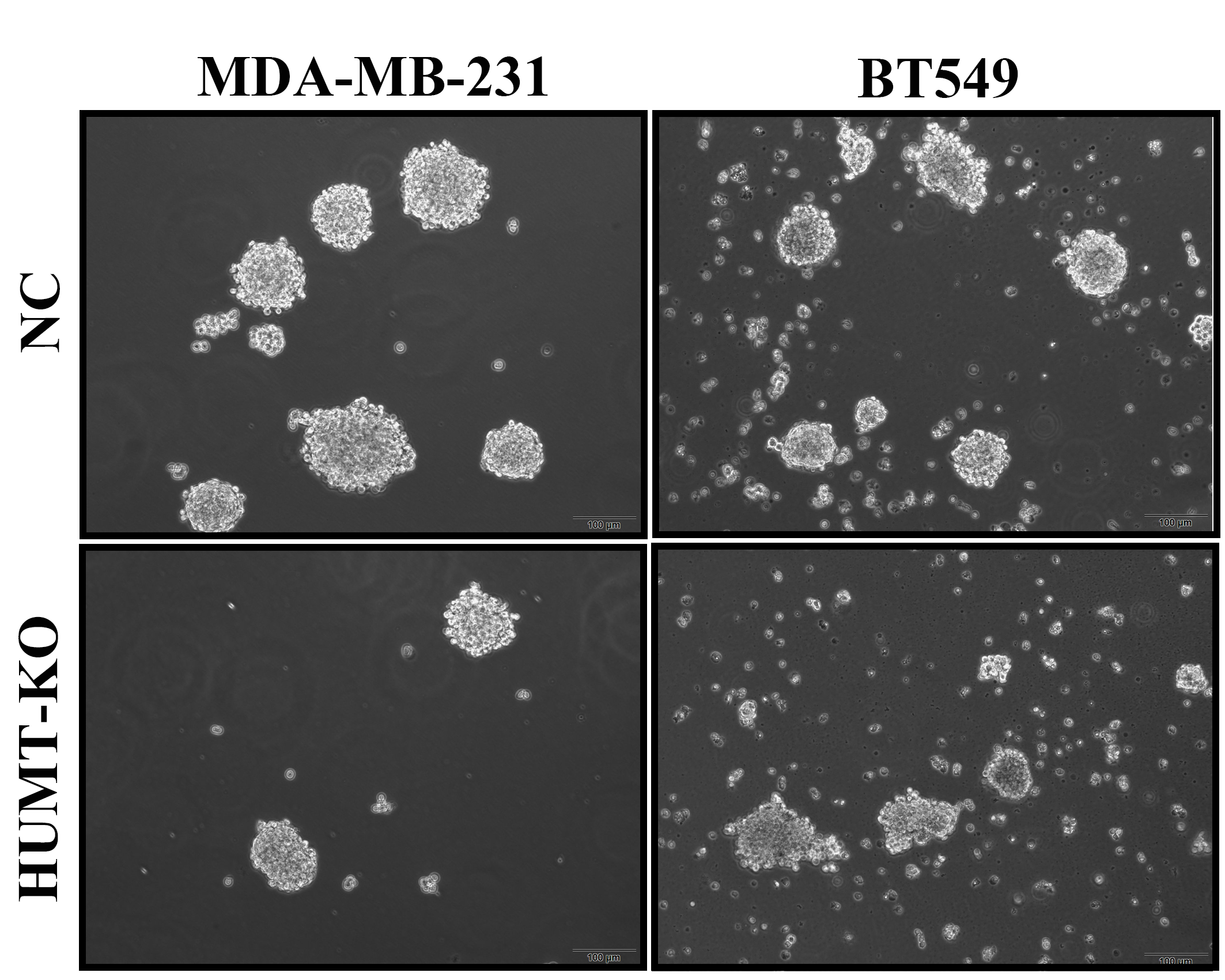

Supplement: Supplementary file 9 — Additional file 9: Figure S9. The tumor spheroids formed by cells in the HUMT-KO or control groups. [file 13045_2020_852_MOESM9_ESM.tif]
