# Supplementary material for: Long non-coding RNA HUMT hypomethylation promotes lymphangiogenesis and metastasis via activating FOXK1 transcription in triple-negative breast cancer
Source: J Hematol Oncol. 2020 Mar 5;13:17. doi: 10.1186/s13045-020-00852-y (PMC7059688; doi:10.1186/s13045-020-00852-y)
Supplement: Supplementary file 10 — Additional file 10: Supplementary Material and Methods. [file 13045_2020_852_MOESM10_ESM.docx]

**Supplementary Materials and Methods**

**Study subjects**

228 triple-negative breast cancer patients between 2005 and 2011 were enrolled in the Sun Yat-sen University Cancer Center (SYSUCC) study population and the following criteria were used for patient selection: 1) breast cancer samples were available and pathologically validated under breast-conserving or modified radical mastectomy; 2) the molecular subtypes were determined by immunohistochemistry (IHC) and Her-2 status was further confirmed by fluorescence in situ hybridization (FISH) if intermediate positive in IHC; 3) complete follow-up data available; 4) free from other malignant cancers; 5) AJCC 7th TNM stage; 6) without neoadjuvant chemotherapy; 7) without distant metastasis. Patients without detailed active follow-up were excluded. Overall survival (OS) was defined as the time from the date of diagnosis to the date of death from any cause or last follow-up. Disease-free survival was defined as the time from the date of diagnosis to the date of first local or distant recurrence or last follow-up. Patients who died without any disease recurrence record were considered censored at the date of death. The follow-up information was acquired from inpatient and outpatient records and follow-up telephone calls. All patients in the SYSUCC cohort have signed written informed consent and the study was approved by the institutional review board of Sun Yat-sen University Cancer Center and conducted under the guidance of Declaration of Helsinki.

For TNBC datasets, the selection criteria were as follows: 1) available for OS data and mRNA expression; 2) available for pathological data of TCGA (The Cancer Genome Atlas) and GEO datasets (GSE76124 and GSE58812), including the pathological diagnosis and molecular subtypes. 159 patients from TCGA, 198 patients from GSE76124 and 107 patients from GSE58812 were enrolled in this study[1, 2].

**In situ hybridization (ISH) and fluorescent in situ hybridization (FISH)**

ISH was performed under the guidance of In Situ Hybridization Kit (Boster Bio-Engineering Company). The paraffin-embedded tissue samples were deparaffinized in xylene and rehydrated via graded ethanol. Then the slides were digested with pepsin for 20 minutes at room temperature. The sections were further prehybridized overnight at 40 ℃. After washes, the sections were incubated in HUMT-specific probe and subsequent biotinylated mouse anti-digoxin antibody and further stained with biotinylated peroxidase. The HUMT expression was further visualized with 3,3’-diaminobenzidine (DAB) substrate (Dako) and counterstained by hematoxylin.

For FISH, MDA-MB-231 and BT549 cells grew on glass cover slips in 24-well plates overnight. The cells were fixed with paraformaldehyde and hybridized with Cy3-labeled probe and Hoechst was used to stain the nuclei. The results were observed under microscope (OLYMPUS IX71).

**Immunohistochemistry (IHC)**

IHC staining was performed in the paraffin-embedded tissue samples. Section slides with tissue were deparaffinized in xylene and rehydrated via graded ethanol (dilution from 100%, 95%, 85% and 75%). Then slides were treated in antigen retrieval buffer (CWBIO) and incubated in 10% hydrogen peroxide for 15 min to block endogenous peroxidase activity before incubating with corresponding primary antibody at under 4°C overnight. After incubated with HRP conjugated secondary antibody for 30 min at room temperature, peroxidase staining was then revealed with 3, 3’-diaminobenzidine (DAB) substrate (Dako). Tissue sections were counterstained by hematoxylin. The stained sections were observed under an optical microscope (NIKON ECLIPSE 80i). CD56-stained NK cells in stromal tissues were counted in 5 random fields and recorded as NK-neg (negative) or NK- pos (positive). The antibodies were listed in the Table S2.

**Hematoxylin-eosin (HE) staining**

The tissues were fixed in 4% formaldehyde, embedded in paraffin, and cut into 4mm sections. After deparaffinized and rehydrated, the sections were dipped in hematoxylin for 5 min, washed with running tap water for 30 min. Then the sections were dyed with eosin for 3 min. Subsequently, the sections were dehydrated in an alcohol gradient and coverslipped. The stained sections were observed under an optical microscope (NIKON ECLIPSE 80i).

**Cell lines and cell culture**

Human breast cancer cell lines and embryo kidney cell line 293T were purchased from the American Type Culture Collection. All cell lines were maintained following standard guidelines. All cell lines were grown without antibiotics in an atmosphere of 5% CO_2_ and 99% relative humidity at 37°C. Cell lines were passaged for fewer than 6 months and were authenticated by short tandem repeat analysis. No mycoplasma infection was found for all cell lines. For tumorsphere culture, NC or HUMT-KO cells (MDA-MB-231 and BT549) were cultured to form spheres in ultra-low attachment dishes (Corning) at a density of 1,000 cells per milliliter for 5 days in DMEM/F12 medium with epidermal growth factor (EGF) (20 ng/ml, Invitrogen), 1% B27 (Invitrogen, Carlsbad, CA), insulin (10 mg/ml, Sigma), and basic fibroblast growth factor (bFGF) (10 ng/ml, Invitrogen).

**RNA isolation and quantitative real-time PCR analysis**

Total RNA of cells and tissues was extracted with TRIzol reagent (Invitrogen). The primer sequences are shown in Supplementary Table S3. RNA levels were determined by quantitative real-time PCR (qRT-PCR) in triplicate on a Bio-Rad CFX96 using the SYBR Green method (Takara). The RNA level was normalized against β-actin RNA using the comparative Ct method.

**Cytoplasmic and nuclear fractionation**

Subcellular fractionation of cells was performed as described previously[3]. Cytoplasmic and nuclear RNAs of MDA-MB-231 and BT549 cells were isolated and purified using the Cytoplasmic and Nuclear RNA Purification Kit (Norgen Biotek).

**Cell viability and colony formation assays**

For cell viability assays of modulated cells, MDA-MB-231 (1.0 × 10^3^ per well) or BT549 (1.5 × 10^3^ per well) cells were seeded in 96-well plates. After a certain time of cultivation, cell viability was measured with the CCK-8 Kit (Dojindo). For colony formation assays, 1000 cells were seeded in 6-well cell culture plates and allowed to grow until visible colonies formed in complete growth medium. Cell colonies were fixed with methanol, stained with crystal violet and counted.

**In vitro invasion and migration assays**

Migration assays were done in 24-well Falcon chamber. The migration assays were conducted with cell number of 2.5 × 10^4^ and without coating the filters with matrigel. The 8-μm pore inserts were used and cells (2.5 × 10^4^) in 200 μl of serum-free medium were added to the upper chamber. A 700μl medium containing 20% fetal bovine serum was added to the lower chamber as chemo-attractant. After appropriate time in an incubator at 37°C, cells that migrated though the filter were fixed with methanol, stained with 0.5% crystal violet, and cell numbers were counted on three random fields.

3D invasion assays were conducted as previously described[4]. Single-cell suspension for hanging drop cultures was prepared by detaching adherent cancer cell cultures of ~70% confluence using a PBS wash followed by exposure to 0.05% trypsin-EDTA solution. Perform a dilution to allow for the seeding of 1000 cells per 20 µl drop of cell culture medium. Pipet 40 drops (5 rows of 8 drops) onto the lid of the 10 cm dish. Invert the lid and place over the culture dish. Incubate the hanging drop cultures at 37℃ for 48 hr to generate spheroids. Mix 100 µl of the basement membrane materials with 100 µl of cold (4℃) type I collagen in a separate pre-chilled tube. The spheroids were collected and combined with the basement membrane materials/type I collagen mixture. The mixture were further incubated in 24-well plates at 37℃, 5% CO_2_ and observed under microscope.

**Endothelial cell tube formation assay**

HLECs were grew in supernant of modulated cancer cells overnight. 10μl of Matrigel was plated to the inner well of ibiTreatμ-Slide plates (ibidi) and incubated for 1h at 37°C. Then, 1×10^4^ HLECs in 50μl were placed onto the top of the Matrigel in each well. After a 4h incubation, tube formation was quantified by measuring the total length of tube structures and/or the number of branch sites in 3 random fields.

**Construction of vectors and transfection**

The sequence of HUMT was cloned into the overexpression vector pcDNA3.1(+) for overexpression in breast cancer cells and termed pcDNA3.1-HUMT. siRNAs targeting YBX1 and FOXK1 was synthesized from IGEbio Technology and the sequences were shown in table S4. For HUMT-KO using CRISPR method, 6 sgRNA targeting HUMT was inserted into the lentiCRISPR v2 vector (Addgene, #52961). The sequence of sgRNAs were listed in table S4. The cells were maintained in complete culture medium containing puromycin and effective clones were selected.

To produce lentivirus, HEK-293T cells were co-transfected with the lentiviral vector described above and packaging vectors psPAX2 and VSVG using EndoFectin Lenti transfection reagent (Genecopoeia). The lentiviral supernant was harvested 48h after transfection and concentrated using Lenti-Pac^TM^ Concentration Solution (Genecopoeia) following the manufacturer’ instructions and further used for cancer cell infection.

For dcas9-CHIP, the 3×Flag-NLS-dCas9-NLS vector was constructed using a pcDNA3.3-TOPO backbone (Addgene) and the sgRNAs was inserted into the EcoRI and BsmBI sites of U6-spgRNA v2.0-CMV-EGFP vector purchased from Obio Technology. All plasmids were verified by DNA sequencing. Transfections were performed using the Lipofectamine 3000 kit (Invitrogen) according to the manufacturer’s instructions.

**RNA immunoprecipitation (RIP)**

Cells (1×10^7^) in a 10 cm plate were harvested and resuspended with RIP lysis buffer after centrifugation. The immunoprecipitation buffer was prepared and mixed with cell lysis. The mixture was incubated rotating at 4℃ overnight. The RNA-protein complex was extracted with magnetic beads and proteinase K was used to digest the protein and purify the RNAs. The purified RNAs were furthered applied to qRT-PCR. The RIP experiment was conducted using Magna RIP™ RNA-Binding Protein Immunoprecipitation Kit (Millipore).

**RNA pulldown**

Briefly, the RNA was labeled by biotinylation using T4 RNA ligase. Biotinylated RNA was incubated with cytoplasmic extract of breast cancer cells at room temperature for 1 hr. Washed nucleic acid compatible streptavidin magnetic beads were added to each binding reaction and further incubated at room temperature for 1 hr. Precipitates were washed for five times and boiled in SDS buffer followed by western blot detection. The procedures described above was conducted using Pierce™ Magnetic RNA-Protein Pull-Down Kit (Thermo Scientific).

**Chromatin immunoprecipitation (CHIP)**

2×10^7^ of cells were trypsinized, washed with PBS twice and collected after centifugation. 1% formaldehyde was used for DNA crosslink at room temperature. The precipitation was washed and applied to lysis buffer to extract the nuclear contents. The DNAs were further fragmented and enriched with anti-YBX1 and IgG antibody. The mixture were further inverse-crosslinked in 65℃ overnight and the enriched DNA was extracted and applied to qRT-PCR. The primers were listed in Table S3. Detailed procedures were depicted in the manufacturer’s guidance of CHIP kit (Bersin Bio).

**dCas9-guided immunoprecipitation (dCas9-CHIP) qPCR**

The dcas9-CHIP of FOXK1 promoter region were performed as previously described to analyze the chromatin occupancy[5]. The sgRNAs and dCas9 vectors were co-transfected into the cells and 2×10^7^ trypsinized cells were crosslinked with formaldehyde at room temperature. The DNA-RNA-protein complex were further fragmented and enriched using an anti-Flag antibody following standard CHIP procedure.

**Western blot analysis**

Protein extracts from cells were prepared using RIPA lysis buffer. Total protein was added to SDS-PAGE and transferred to PVDF membrane (Millipore). Antibody against YBX1, FOXK1, HIF-α, mTOR, p-mTOR, p-Akt, Akt, VEGFC, CD56, β-Actin, GAPDH was used. Membrane was incubated with primary antibody at 4°C overnight and subsequent secondary antibody at room temperature for 1h. The blots were further visualized with Immobilon Western Chemiluminescent HRP Substrate (Beyotime). The antibodies are listed in the Table S2 (Additional file 12).

**Animal experiments**

Female BALB/c nude mice, aged 3-4 weeks, were purchased from the Beijing Vital River Laboratories Animal Technology and used for the experiments after adapting to local conditions for 1 week.

To examine the effects of HUMT on growth of implanted tumors, a total of 5×10^6^ of luciferase-labeled MDA-MB-231 cells with HUMT-KO were injected subcutaneously in the fat pads of mice with 1:1 basement membrane matrix (Matrigel, Corning). When a tumor was palpable, it was measured every 4 days and the volume was calculated according to the formula volume = length × width^2^ /2. The mice were sacrificed at the end of experiment and xenografts were stripped, weighted and photographed. For PDX study, the xenografts were transplanted as previously described and we perfomed intratumoral injection with ASO[6]. Tumour weights were examined.

To test the effect of HUMT on cancer cell metastasis, luciferase-labeled 231LNM3 cells were transfected and used. A number of 2.5×10^6^ modulated 231LNM3 cells was injected with 1:1 basement membrane matrix (Matrigel, Corning) into the fat pads of mice and observed for lymph node metastasis. A number of 1×10^6^ modulated 231LNM3 cells was injected through the tail vein to construct lung metastasis model. At the end of experiment, after intraperitoneal injection of 2.0 mg luciferin (Promega) for 10 minutes, the mice was sacrificed and dissected. The lung metastases were detected using a Living Image® system (Perkin Elmer) and the lung nodules were counted. All procedures were performed in accordance with relevant institutional and national guidelines and regulations.

**Bioinformatics analysis**

The TCGA dataset was obtained from TCGA database using gdc-client. The mRNA expression matrices of GSE76124 and GSE58812 were downloaded from the GEO dataset[2, 7]. The CHIP-seq datasets were download from ENCODE database (https://www.encodeproject.org) and analyzed under the guidance. Bedtools and homer were further used for sequence mapping and visualization[8, 9]. For tumor infiltrating immune cells prediction, the expression matrices were uploaded to the CIBERSORT and calculated according to LM22 signature with 1000 permutations[10]. The valid samples were selected using a *P* value of <0.05. All datasets and tools were listed in table S5 and S6[11-15].

**Reference**

1. Burstein MD, Tsimelzon A, Poage GM, Covington KR, Contreras A, Fuqua SA, Savage MI, Osborne CK, Hilsenbeck SG, Chang JC, et al: **Comprehensive genomic analysis identifies novel subtypes and targets of triple-negative breast cancer.** *Clin Cancer Res* 2015, **21:**1688-1698.

2. Jezequel P, Loussouarn D, Guerin-Charbonnel C, Campion L, Vanier A, Gouraud W, Lasla H, Guette C, Valo I, Verriele V, Campone M: **Gene-expression molecular subtyping of triple-negative breast cancer tumours: importance of immune response.** *Breast Cancer Res* 2015, **17:**43.

3. Huang X, Xie X, Liu P, Yang L, Chen B, Song C, Tang H, Xie X: **Adam12 and lnc015192 act as ceRNAs in breast cancer by regulating miR-34a.** *Oncogene* 2018, **37:**6316-6326.

4. Li T, Hu PS, Zuo Z, Lin JF, Li X, Wu QN, Chen ZH, Zeng ZL, Wang F, Zheng J, et al: **METTL3 facilitates tumor progression via an m(6)A-IGF2BP2-dependent mechanism in colorectal carcinoma.** *Mol Cancer* 2019, **18:**112.

5. Campbell AE, Shadle SC, Jagannathan S, Lim JW, Resnick R, Tawil R, van der Maarel SM, Tapscott SJ: **NuRD and CAF-1-mediated silencing of the D4Z4 array is modulated by DUX4-induced MBD3L proteins.** *Elife* 2018, **7**.

6. Zhu P, Wu J, Wang Y, Zhu X, Lu T, Liu B, He L, Ye B, Wang S, Meng S, et al: **LncGata6 maintains stemness of intestinal stem cells and promotes intestinal tumorigenesis.** *Nat Cell Biol* 2018, **20:**1134-1144.

7. Dent R, Trudeau M, Pritchard KI, Hanna WM, Kahn HK, Sawka CA, Lickley LA, Rawlinson E, Sun P, Narod SA: **Triple-negative breast cancer: clinical features and patterns of recurrence.** *Clin Cancer Res* 2007, **13:**4429-4434.

8. Quinlan AR, Hall IM: **BEDTools: a flexible suite of utilities for comparing genomic features.** *Bioinformatics* 2010, **26:**841-842.

9. Heinz S, Benner C, Spann N, Bertolino E, Lin YC, Laslo P, Cheng JX, Murre C, Singh H, Glass CK: **Simple combinations of lineage-determining transcription factors prime cis-regulatory elements required for macrophage and B cell identities.** *Mol Cell* 2010, **38:**576-589.

10. Newman AM, Liu CL, Green MR, Gentles AJ, Feng W, Xu Y, Hoang CD, Diehn M, Alizadeh AA: **Robust enumeration of cell subsets from tissue expression profiles.** *Nat Methods* 2015, **12:**453-457.

11. Ritchie ME, Phipson B, Wu D, Hu Y, Law CW, Shi W, Smyth GK: **limma powers differential expression analyses for RNA-sequencing and microarray studies.** *Nucleic Acids Res* 2015, **43:**e47.

12. McCarthy DJ, Chen Y, Smyth GK: **Differential expression analysis of multifactor RNA-Seq experiments with respect to biological variation.** *Nucleic Acids Res* 2012, **40:**4288-4297.

13. Robinson MD, McCarthy DJ, Smyth GK: **edgeR: a Bioconductor package for differential expression analysis of digital gene expression data.** *Bioinformatics* 2010, **26:**139-140.

14. Wang L, Park HJ, Dasari S, Wang S, Kocher JP, Li W: **CPAT: Coding-Potential Assessment Tool using an alignment-free logistic regression model.** *Nucleic Acids Res* 2013, **41:**e74.

15. Li LC, Dahiya R: **MethPrimer: designing primers for methylation PCRs.** *Bioinformatics* 2002, **18:**1427-1431.
