# Supplementary material for: Long non-coding RNA HUMT hypomethylation promotes lymphangiogenesis and metastasis via activating FOXK1 transcription in triple-negative breast cancer
Source: J Hematol Oncol. 2020 Mar 5;13:17. doi: 10.1186/s13045-020-00852-y (PMC7059688; doi:10.1186/s13045-020-00852-y)
Supplement: Supplementary file 11 — Additional file 11: Table S1. [file 13045_2020_852_MOESM11_ESM.docx]

**Table S1. Association between HUMT and the clinicopathological characteristics of TNBC.**

| Variables | Cases(n=228) | HUMT | |  | *P* value |
| --- | --- | --- | --- | --- | --- |
|  |  | Low  No.(*N*=96) | High  No.(*N*=132) |  |  |
| Age(years) |  |  |  |  |  |
| ≤40 | 58 | 25(43.1%) | 33(56.9%) |  | 0.858 |
| >40 | 170 | 71(41.8%) | 99(58.2%) |  |  |
| Menopause |  |  |  |  |  |
| No | 137 | 61(44.5%) | 76(55.5%) |  | 0.364 |
| Yes | 91 | 35(38.5%) | 56(61.5%) |  |  |
| T stage |  |  |  |  |  |
| T1-2 | 206 | 94(45.6%) | 112(54.4%) |  | <0.001^*^ |
| T3-4 | 22 | 2(9.1%) | 20(90.9%) |  |  |
| N stage |  |  |  |  |  |
| N0 | 111 | 89(80.2%) | 22(19.8%) |  | <0.001^*^ |
| N1-3 | 117 | 7(6.0%) | 110(94.0%) |  |  |
| TNM stage |  |  |  |  |  |
| Ⅰ-Ⅱ | 174 | 92(52.9%) | 82(47.1%) |  | <0.001^*^ |
| Ⅲ-Ⅳ | 54 | 4(7.4%) | 50(92.6%) |  |  |
| Grade |  |  |  |  | 0.100^a^ |
| G1 | 3 | 3(100%) | 0(0%) |  |  |
| G2 | 137 | 59(43.1%) | 78(56.9%) |  |  |
| G3 | 88 | 34(38.6%) | 54(61.4%) |  |  |
| BMI |  |  |  |  | 0.929 |
| <23.9 | 148 | 62(41.9%) | 86(58.1%) |  |  |
| ≥23.9 | 80 | 34(42.5%) | 46(57.5%) |  |  |

^*^*P* < 0.05, statistically significant
