# Supplementary material for: Long non-coding RNA HUMT hypomethylation promotes lymphangiogenesis and metastasis via activating FOXK1 transcription in triple-negative breast cancer
Source: J Hematol Oncol. 2020 Mar 5;13:17. doi: 10.1186/s13045-020-00852-y (PMC7059688; doi:10.1186/s13045-020-00852-y)
Supplement: Supplementary file 12 — Additional file 12: Table S2. [file 13045_2020_852_MOESM12_ESM.docx]

**Table S2. Antibodies and biological samples in this study.**

| Antibody | Source | Application |
| --- | --- | --- |
| β-actin | Cell Signaling Technology | 1:2000 for WB |
| YBX1 | Zenbio | 1:1000 for WB;1:50 for RIP and CHIP |
| FOXK1 | Cell Signaling Technology | 1:1000 for WB |
| HIF1α | Zenbio | 1:1000 for WB |
| VEGFC | Proteintech | 1:1000 for WB |
| Akt | Cell Signaling Technology | 1:1000 for WB |
| p-Akt(S473) | Cell Signaling Technology | 1:1000 for WB |
| mTOR | Cell Signaling Technology | 1:1000 for WB |
| p-mTOR | Cell Signaling Technology | 1:1000 for WB |
| GAPDH | MERCK | 1:2000 for WB |
| CD56 | ZSGB | Ready to use |
| Flag | Proteintech | 3μg for dcas9IP |
| Anti-Rabbit IgG (H+L), HRP Conjugate | Promega | 1:3000 for WB |
| Anti-Mouse IgG (H+L), HRP Conjugate | Promega | 1:3000 for WB |

| Biological samples | Source | Aplication |
| --- | --- | --- |
| Clinical breast cancer tissue samples | This paper (Sun Yat-sen University Cancer Center) | ISH, qRT-PCR, WB |
