# Supplementary material for: Long non-coding RNA HUMT hypomethylation promotes lymphangiogenesis and metastasis via activating FOXK1 transcription in triple-negative breast cancer
Source: J Hematol Oncol. 2020 Mar 5;13:17. doi: 10.1186/s13045-020-00852-y (PMC7059688; doi:10.1186/s13045-020-00852-y)
Supplement: Supplementary file 13 — Additional file 13: Table S3. [file 13045_2020_852_MOESM13_ESM.docx]

**Table S3. Primers sequence**

**Primers for qRT-PCR detection**

| HUMT | Forward | CATCATACAAGGTGGAGAAGAG |
| --- | --- | --- |
|  | Reverse | CAATGTCTGATCTCCAGCC |
| U2 | Forward | TCGGCACCGAGAAAGACAAA |
|  | Reverse | CGTCTGTGCACTGTTTTGGG |
| β-actin | Forward | AATCGTGCGTGACATTAAGGAG |
|  | Reverse | ACTGTGTTGGCGTACAGGTCTT |
| FOXK1 | Forward | CATTACCCCTACTACCGGACG |
|  | Reverse | GTAACGGTTCAAAGAGAGGTTGT |
| YBX1 | Forward | CCAGGAAGTACCTTCGCAGTG |
|  | Reverse | AGGACCCCTACGACGTGGAT |

**Primers for qRT-PCR after CHIP.**

| -250~-400bp | Forward | CTGGGGACTTTACGGGTCGG |
| --- | --- | --- |
|  | Reverse | ACGACTCCAGTAGGCAGGGAA |
| -401~-550bp | Forward | GGGGGTTGGGATTTCACATT |
|  | Reverse | TACTAAAACGGTGGCGTGAC |
| -551~-700bp | Forward | AGGACGGAGACGGAGTGTTC |
|  | Reverse | CGGTTGTATCGTTCTGGGACA |
| -701~-850bp | Forward | GAGCTGGAGGGTTTCGTGAC |
|  | Reverse | AGTCCCTACCTAGAGTTCACCA |
