# Supplementary material for: Long non-coding RNA HUMT hypomethylation promotes lymphangiogenesis and metastasis via activating FOXK1 transcription in triple-negative breast cancer
Source: J Hematol Oncol. 2020 Mar 5;13:17. doi: 10.1186/s13045-020-00852-y (PMC7059688; doi:10.1186/s13045-020-00852-y)
Supplement: Supplementary file 14 — Additional file 14: Table S4. [file 13045_2020_852_MOESM14_ESM.docx]

**Table S4. siRNAs and sgRNAs sequence**

**Sequence of siRNAs**

| Non-targeting | siControl#1 | 5'-UGGUUUACAUGUCGACUAAUU |
| --- | --- | --- |
|  | siControl#2 | 5'-UGGUUUACAUGUUGUGUGAUU |
| FOXK1 | siFOXK1#1 | 5'-GUUCACGUCGCUCUAUCACUU |
|  | siFOXK1#2 | 5'-AGGCUGGCAGAAUUCUAUCUU |
|  | siFOXK1#3 | 5'-GAAUUCUAUCCGGCACAACUU |
| YBX1 | siYBX1#1 | 5'-UGACACCAAGGAAGAUGUAUU |
|  | siYBX1#2 | 5'-GUGAGAGUGGGGAAAAGAAUU |
|  | siYBX1#3 | 5'-UAACAGUUUAGAUGCUACCUU |

**Sequence of sgRNAs**

| HUMT | sgRNA#1 | AAGAATGGTGAAGCGCACGA |
| --- | --- | --- |
|  | sgRNA#2 | GAATGGTGAATAGCTACCCC |
|  | sgRNA#3 | GGCTGTTACAAACAAGCTGC |
|  | sgRNA#4 | AGCGACTTTAAAACGCAAAT |
|  | sgRNA#5 | GCCCGGGGATTGGTCCGGGA |
|  | sgRNA#6 | CGAAAGAGAGCCGCGCCTGC |
| FOXK1(dCas9-CHIP) | sgRNA#1 | GTATGATGACCTTTAATCTG |
|  | sgRNA#2 | TGGGCGTGGTGGCACTTGTG |
|  | sgRNA#3 | GTCCCTTTCTCCAATGACCC |
