# Supplementary material for: Long non-coding RNA HUMT hypomethylation promotes lymphangiogenesis and metastasis via activating FOXK1 transcription in triple-negative breast cancer
Source: J Hematol Oncol. 2020 Mar 5;13:17. doi: 10.1186/s13045-020-00852-y (PMC7059688; doi:10.1186/s13045-020-00852-y)
Supplement: Supplementary file 15 — Additional file 15: Table S5. [file 13045_2020_852_MOESM15_ESM.docx]

**Table S5. Datasets used in this study.**

| Dataset | Source | Indentifier |
| --- | --- | --- |
| The Cancer Genome Atlas | https://portal.gdc.cancer.gov | BRCA |
| Cancer Cell Line Encyclopedia | https://portals.broadinstitute.org/ccle | CCLE |
| KM Plotter | http://kmplot.com/analysis | None |
| Comprehensive genomic analysis identify novel subtypes and targets of triple-negative breast cancer | https://www.ncbi.nlm.nih.gov/geo/query/acc.cgi | GSE76124 |
| Gene-expression molecular subtyping of triple-negative breast cancer tumours: importance of immune response | https://www.ncbi.nlm.nih.gov/geo/query/acc.cgi | GSE58812 |
| PARCLIP analysis of YBX1, ELAVL1 and ALYREF to detect their binding targets | https://www.ncbi.nlm.nih.gov/geo/query/acc.cgi | GSE133620 |
| CHIP-seq of YBX1 in K562, HepG2 and GSM12878 | https://www.encodeproject.org | YBX1 |
| bc-GenExMiner v4.3 | http://bcgenex.centregauducheau.fr/BC-GEM/GEM-Citation.php | BRCA |
