# Supplementary material for: Long non-coding RNA HUMT hypomethylation promotes lymphangiogenesis and metastasis via activating FOXK1 transcription in triple-negative breast cancer
Source: J Hematol Oncol. 2020 Mar 5;13:17. doi: 10.1186/s13045-020-00852-y (PMC7059688; doi:10.1186/s13045-020-00852-y)
Supplement: Supplementary file 16 — Additional file 16: Table S6. [file 13045_2020_852_MOESM16_ESM.docx]

**Table S6. Related software and algorithms used in this study.**

| Software and Algorithms | Source | Indentifier |
| --- | --- | --- |
| edgeR | Robinson et al., 2010 | https://bioconductor.org/packages/release/bioc/html/edgeR.html |
| limma | Ritchie, M.E. et al., 2015 | https://bioconductor.org/packages/release/bioc/html/limma.html |
| homer | Heinz S et al., 2010 | http://homer.ucsd.edu/homer/ |
| Cibersort | Aaron M Newman et al., 2015 | https://cibersort.stanford.edu |
| GraphPad 7 | GraphPad | https://www.graphpad.com/scientific-software/prism/ |
| Image J | National Institutes of Health | https://imagej.nih.gov/ij/ |
| MethPrimer | Li LC et al., 2002 | http://www.urogene.org/cgi-bin/methprimer/methprimer.cgi |
| Medcalc | MedCalc Software | https://www.medcalc.org/index.php |
| CPAT | Wang, L. et al., 2013 | http://lilab.research.bcm.edu/cpat/ |
| ORFfinder | National Center for Biotechnology Information | https://www.ncbi.nlm.nih.gov/orffinder/ |
